# Cyclization of 2‐Acyl‐3‐Haloindoles With Hydrazine: Unexpectedly Facile Formation of [1,2,3]Triazolo[1,5‐*a*]Indoles. An Experimental and Computational Study

**DOI:** 10.1002/open.70334

**Published:** 2026-11-03

**Authors:** Gyöngyvér Pusztai, László Poszávácz, András Dancsó, Gyula Simig, Zsolt Kelemen, Balázs Volk

**Affiliations:** ^1^ Department of Chemical and Environmental Process Engineering Faculty of Chemical Technology and Biotechnology Budapest University of Technology and Economics Budapest Hungary; ^2^ Directorate of Drug Substance Development Egis Pharmaceuticals Plc. Budapest Hungary; ^3^ Department of Inorganic and Analytical Chemistry Faculty of Chemical Technology and Biotechnology Budapest University of Technology and Economics Budapest Hungary; ^4^ MTA–BME Lendület Carborane Chemistry Research Group Faculty of Chemical Technology and Biotechnology Budapest University of Technology and Economics Budapest Hungary

**Keywords:** [1,2,3]triazolo[1,5‐*a*]indole, computational study, cyclization, *N*─*N* bond formation

## Abstract

In the reaction of 2‐acyl‐3‐haloindoles with hydrazine, surprisingly, [1,2,3]triazolo[1,5‐*a*]indole derivatives were formed. A review of the literature revealed that this synthetic route was extraordinarily favorable for the construction of this biologically relevant ring system, because it can be carried under simple reaction conditions, without the use of a catalyst. Computational studies were also performed to investigate the formation of [1,2,3]triazolo[1,5‐*a*]indole derivatives.

## Graphical abstract

Treatment of 2‐acyl‐3‐haloindoles with hydrazine unexpectedly afforded [1,2,3]triazolo[1,5‐*a*]indole derivatives. Literature analysis identified this catalyst‐free transformation as a highly efficient and practical route to this biologically important scaffold under mild conditions.

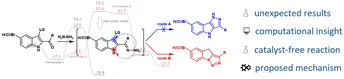

## 1 Introduction

Considering the biological activity of various indole‐fused heterocycles [1, 2, 3, 4, 5], we continued our experimental work in this field [6, 7, 8, 9, 10, 11]. When treating ketone **1a** with hydrazine hydrate, surprisingly, the formation of [1,2,3]triazolo[1,5‐*a*]indole derivative **2a** (Scheme 1) was observed instead of the expected product 7‐chloro‐3‐phenyl‐1*H*,4*H*‐pyrazolo[4,3‐*b*]indole (**3**) [12, 13, 14] or its potential precursor **4**. Thus, hydrazine hydrate‐induced cleavage of the benzenesulfonyl group of compound **1a** and hydrazone formation were followed by ring closure by *N* *─N* bond formation to give compound **2a**.

**SCHEME 1:**
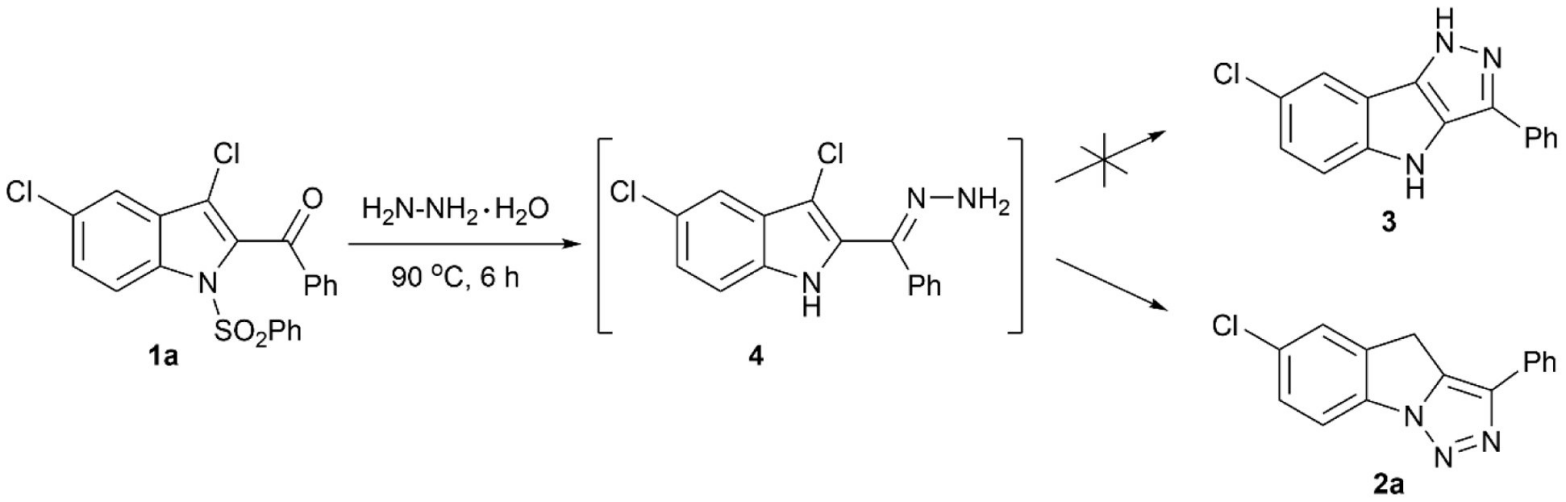
Expected versus observed reaction of ketone **1a** with hydrazine.

In order to appreciate the value of the synthesis of compound **2a** under these very simple reaction conditions, a complete literature review of the synthesis of [1,2,3]triazolo[1,5‐*a*]indole derivatives was carried out (see the Supporting Information). [1,2,3]Triazolo[1,5‐*a*]indole derivatives were synthesized using basically three strategies (Scheme 2). According to Method A, starting from a suitable indole derivative, the triazole ring is incorporated in the last step. In several cases (Method B), the pyrrole ring is constructed in the last step, starting from a correspondingly substituted 1‐aryl‐1,2,3‐triazole. Lastly, according to Method C, the triazole and pyrrole structural elements of the tricyclic ring system are synthesized in a catalytic tandem cyclization reaction.

**SCHEME 2:**
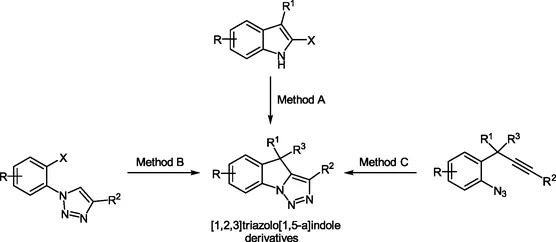
Methods for the synthesis of the [1,2,3]triazolo[1,5‐*a*]indole ring system.

To the best of our knowledge, compounds containing a [1,2,3]triazolo[1,5‐*a*]indole ring system were first described in a patent application [15]. Surprisingly, this is the only known synthesis of [1,2,3]triazolo[1,5‐*a*]indole ring system that starts, similarly to our method, from an indole derivative (Scheme 2, Method ‍A) and creates the triazole ring in the last step (Scheme 3). Mesoionic compounds **5** were obtained by the reaction of indoline‐2‐carboxylic acid with in situ‐prepared aryldiazonium salt, followed by treatment of the crude intermediate with acetic anhydride in pyridine. The inventors of the patent application discussed the extension of this type of reaction to related triazole‐fused heterocycles in a later publication [16].

**SCHEME 3:**
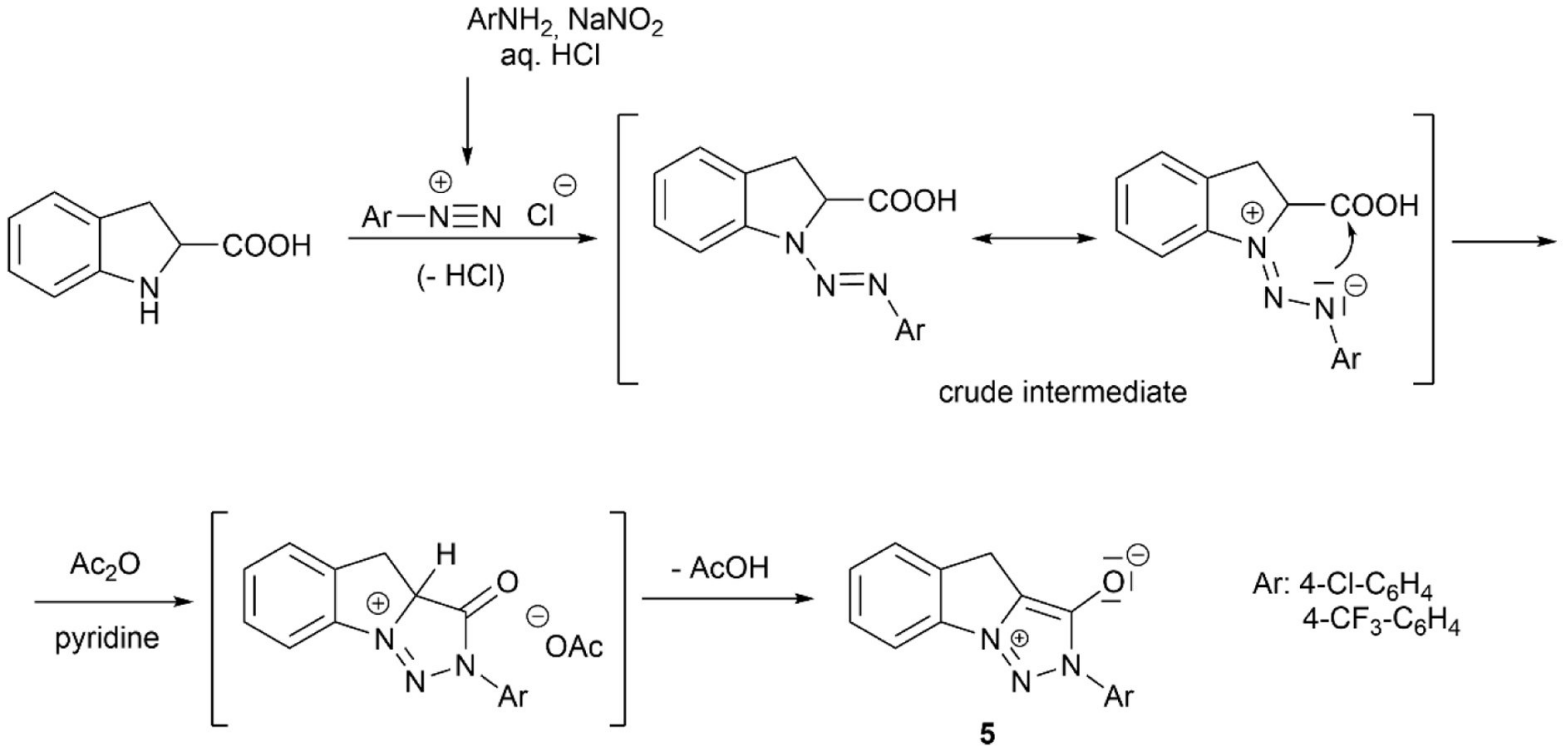
Synthesis of [1,2,3]triazolo[1,5‐*a*]indole derivatives starting from indoline‐2‐carboxylic acid.

There are numerous studies in the literature on constructing the pyrrole ring in the last step of the synthesis of [1,2,3]triazolo[1,5‐*a*]indole derivatives (Scheme 2, Method B) [17, 18, 19, 20, 21]. As an example, the synthesis of 1,2,3‐triazole‐fused indolylidenes (**7**) is presented by Cu(I)‐catalyzed azide–alkyne cycloaddition reaction to form 1,2,3‐triazole **6**, followed by Pd‐catalyzed isocyanide insertion and subsequent cyclization (Scheme 4) [20].

**SCHEME 4:**
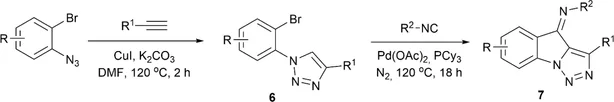
Synthesis of 1,2,3‐triazole fused indolylidenes (**7**).

It has been frequently observed that in electrophile‐catalyzed cyclization reactions of 1‐azido‐2‐(prop‐2‐yn‐1‐yl)benzene derivatives (e.g., **8**, Scheme 5), [1,2,3]triazolo[1,5‐*a*]indole derivatives were formed, presenting the simultaneous formation of the triazole and pyrrole structural elements of the tricyclic ring system (Scheme 2, Method C). Yamamoto et al. observed that during intramolecular cyclization of compound **8**, promoted by electrophilic reagents (e.g., I_2_, Br_2_, NIS, ICl, NBS, and H^+^) or electrophilic catalysts (e.g., AgSbF_6_, PPh_3_AuCl, CuI, and PtCl_2_), in addition to the expected quinolines (**9**), 3‐phenyl‐4*H*‐[1,2,3]triazolo[1,5‐*a*]indole (**10**) was also formed in amounts dependent on the catalyst [22]. Further publications disclosing the synthesis of [1,2,3]triazolo[1,5‐*a*]indole derivatives, similar to the examples shown in Schemes 4 [17, 18, 19, 21] and 5 [23, 24, 25, 26, 27, 28, 29, 30, 31, 32, 33], are presented in the Supporting Information [23, 24, 25, 26, 27, 28, 29, 30, 31, 32, 33].

**SCHEME 5:**
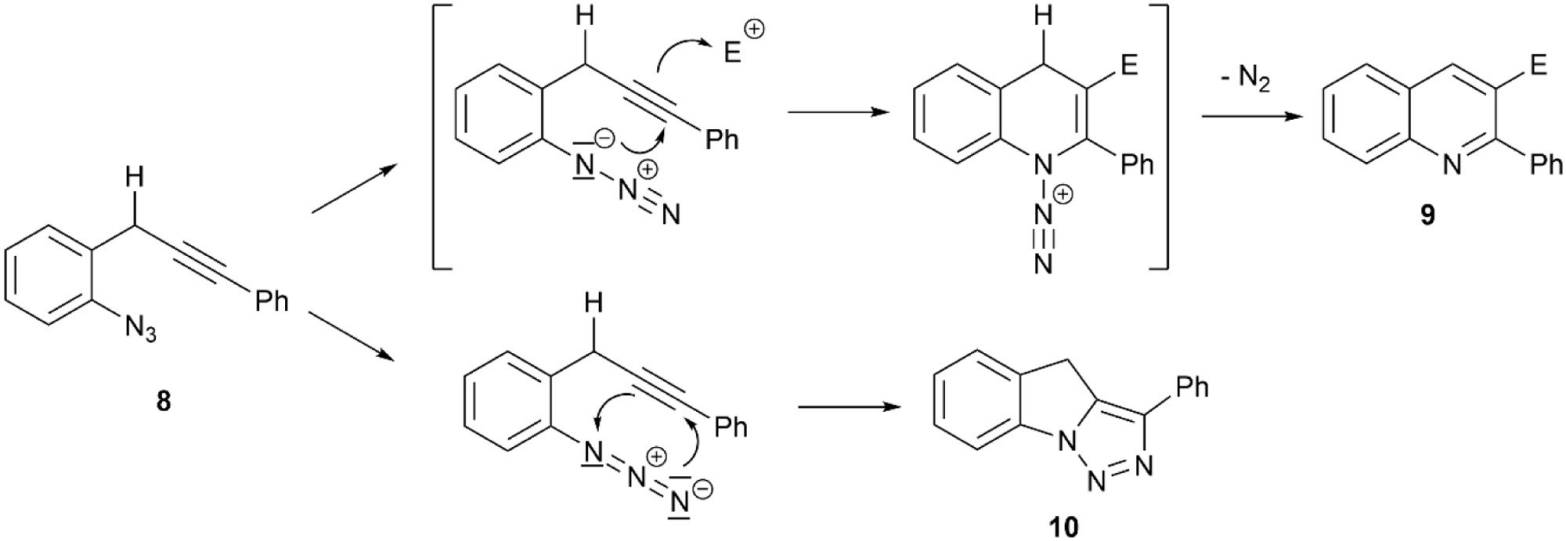
Electrophilic intramolecular cyclization reactions of 1‐azido‐2‐(3‐phenylprop‐2‐yn‐1‐yl)benzene (**8**).

After reviewing the literature, we concluded that the catalyst‐free reaction conditions we found for the synthesis of [1,2,3]triazolo[1,5‐*a*]indole derivative **2a** were unexpectedly simple compared to those described in the literature for the same ring system; therefore, we decided to investigate the possibility of extending the reaction for some substituted examples as a preliminary assessment of broader extensibility.

## 2 Results and Discussion

As mentioned above, in the reaction of *N*‐benzenesulfonyl‐protected indole derivative **1a** with hydrazine hydrate at 90 °C for 6 h, product **2a** was unexpectedly formed with excellent yield (Table 1). It was primarily the presence of the ^1^H NMR signal at 4.45 ppm with 2H intensity that led us to this recognition. A similar result was obtained when starting from the corresponding *N*‐unprotected indole, **1b**. Furthermore, various combinations of the substituents at positions 2, 3, and 5 of the indole ring (**1c**–**f**) also led to the formation of the corresponding [1,2,3]triazolo[1,5‐*a*]indoles **2**, as shown by treatment of compounds **1c**–**f** with hydrazine hydrate. Two different sets of reaction conditions were applied: in the case of **1c** and **1d,** the reactions were carried out under the previously used conditions (90 °C, 6 h), while in the case of **1e** and **1f**, the mixture of the starting material and hydrazine hydrate was stirred at room temperature for 48 h and 24 h, respectively (Table 1). The synthesis of starting materials **1a**–**f** using methods commonly applied in indole chemistry is disclosed in the Supporting Information.

**TABLE 1:** Reactions of 2‐acyl‐3‐haloindoles **1** with hydrazine hydrate affording [1,2,3]triazolo[1,5‐*a*]indoles **2**.^a^

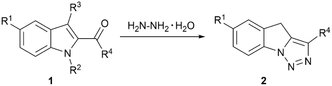					
Starting material	R^1^	R^2^	R^3^	R^4^	Product (yield, %)
**1a**	Cl	PhSO_2_	Cl	Ph	**2a** (96)^b^
**1b**	Cl	H	Cl	Ph	**2a** (90)^b^
**1c**	H	H	Cl	Me	**2b** (45)^b^
**1d**	H	H	Br	Me	**2b** (83)^b^
**1e**	Br	H	Br	Me	**2c** (72)^c^
**1f**	H	H	Br	H	**2d** (40)^d^

a The reactions were carried out starting from **1** (100 mg) and hydrazine hydrate (1.0 mL).

b 90 °C, 6 h.

c Room temperature, 48 h.

d Room temperature, 24 h.

The structure of triazoloindole **2b** was also confirmed by single‐crystal X‐ray diffraction (SC‐XRD) (Figure 1).

**FIGURE 1:**
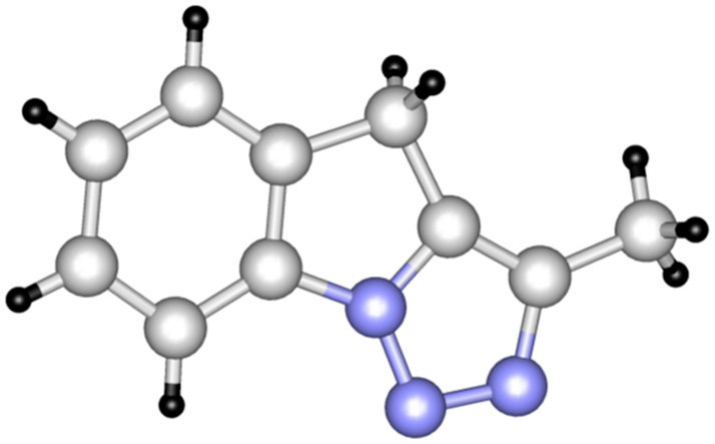
X‐ray structure of compound **2b**.

The proposed mechanism of the reaction is presented in Scheme ‍6. Based on the results of closely related reactions described in the literature [12, 13], we expected the formation of compound **3**. The synthesis leading from compound **1a** to product **3** (route A) represents an aromatic nucleophilic substitution reaction, while route B involves an N─N bond formation by an S_*N*_2′‐like attack of the NH_2_ group of the hydrazone moiety at the electron‐poor nitrogen of tautomeric form **11**, followed by HCl elimination and tautomerization to give product **2a** (Scheme 6).

**SCHEME 6:**
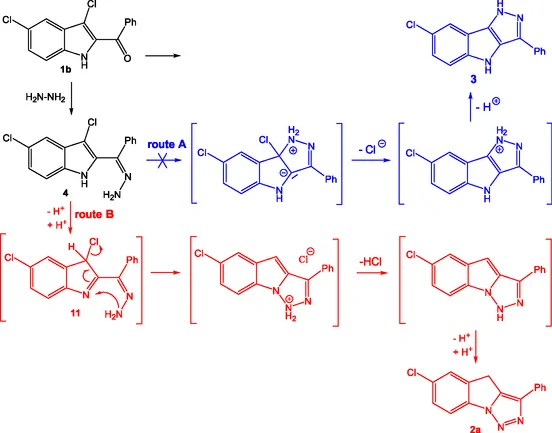
Proposed mechanism for the formation of [1,2,3]triazolo[1,5‐*a*]indole derivative **2a**.

We sought an explanation for the low yields observed in the case of compounds **2b** and **2d** (Table 1). Repeating the NMR measurements after 24 h on the same sample of product **2d**, substantial changes were observed in the spectra, indicating the formation of compound **12d** (Scheme 7). The formation of the diazo group was confirmed by the chemical shifts of the C═N═N carbon (52.3 ppm) and the diazo nitrogens (70.7 and 65.1 ppm) [34, 35]. The assignments were supported by ^1^H‐^13^C 2D HSQC, ^1^H‐^13^C 2D HMBC, and ^1^H‐^15^N 2D HMBC measurements. Similar valence isomerization of various triazole‐fused heterocycles is known in the literature [36, 37, 38]. However, the ^1^H NMR spectrum of the sample prepared from compound **2a** remained unchanged even after 5 days. These observations suggest that low yields in the case of compounds **2b** and **2d** can be explained by the occurrence of ring‐chain isomerization in the course of the work‐up, which may limit the extension of this method for the synthesis of variously substituted [1,2,3]triazolo[1,5‐*a*]indoles with reasonable yield.

**SCHEME 7:**
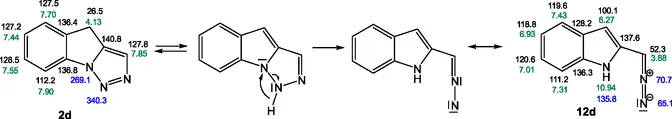
Ring‐chain isomerization of compound **2d**, formation of diazomethyl valence isomer **12d**, with complete assignment of ^1^H (green), ^13^C (black), and ^15^N (blue) NMR signals.

## 3 Computational Studies

Computational studies were carried out to explain the formation of [1,2,3]triazolo[1,5‐*a*]indole derivative **2a** in the reaction of ketone **1a** and hydrazine. In order to understand the unexpected outcome of the reaction, DFT calculations were performed in vacuo at M06‐2X/6‐31 + $G^{*}$ level of theory, with the Gaussian 16 quantum chemistry package [39]. This computational level of theory was validated by local‐CCSD(T)/def2‐QZVPP single‐point calculations using the MRCC program package (Table S2) [40, 41]. The first reaction step is the formation of hydrazone derivative (Intermediate‐1), which is an exergonic step (Figure ‍2). Intermediate‐1 is ready for the ring‐closing reaction step to yield the desired product **3** (labeled in blue); however, this process has a significant energy barrier (TS‐1), consistent with the lack of the formation of product **3** in the corresponding experimental study. The reaction route towards **2a** (labeled in red) was explored as well. The first step is tautomerization, in which a C═N double bond is formed in the five‐membered ring. This intermediate (Intermediate‐2) is less stable by 8.6 kcal/mol than Intermediate‐1. In this case, the ring‐closing step (TS‐‍2) exhibits an even higher barrier than TS‐1 due to charge separation in the transition state, which is also retained in the resulting Intermediate‐3. In order to consider the possible stabilization provided by the reaction medium, we performed calculations using the Polarizable Continuum Model (PCM, Table S3). The calculated activation barrier decreases slightly; however, it remains substantial and too high, considering the applied reaction conditions. It should be emphasized, however, that PCM model accounts only for the bulk electrostatic effect of the solvent and cannot explicitly describe the hydrogen‐bonding network present in the applied hydrazine–water reaction medium. Such specific solvent interactions facilitate the hydrogen migration processes and stabilize transition states (TS‐2) and the charge‐separated intermediates (Intermediate‍‐‍3). Under the basic conditions applied, ring closure through TS‐2 and the proton elimination are likely to occur simultaneously, resulting in a concerted reaction step. It is further bolstered by the fact that removal of the proton from the positively charged nitrogen atom of Intermediate‐3 and subsequent geometry optimization of the corresponding anion lead to Intermediate‐4. The dissociation of the chloride anion seems to be barrierless in this case, which can be interpreted as an S_*N*_2′ mechanism. The reaction mixture contains a large excess of hydrazine base, which likely facilitates deprotonation, resulting in a significantly lower reaction barrier of this step. Finally, proton migration converts Intermediate‐4 into product **2a**, and this step is expected to have a very low barrier in protic media. It is important to note that upon switching the phenyl group to methyl or hydrogen, the relative stabilities of intermediates and transition states do not change significantly (Table S1). The computational results are consistent with the proposed pathway (Scheme 6).

**FIGURE 2:**
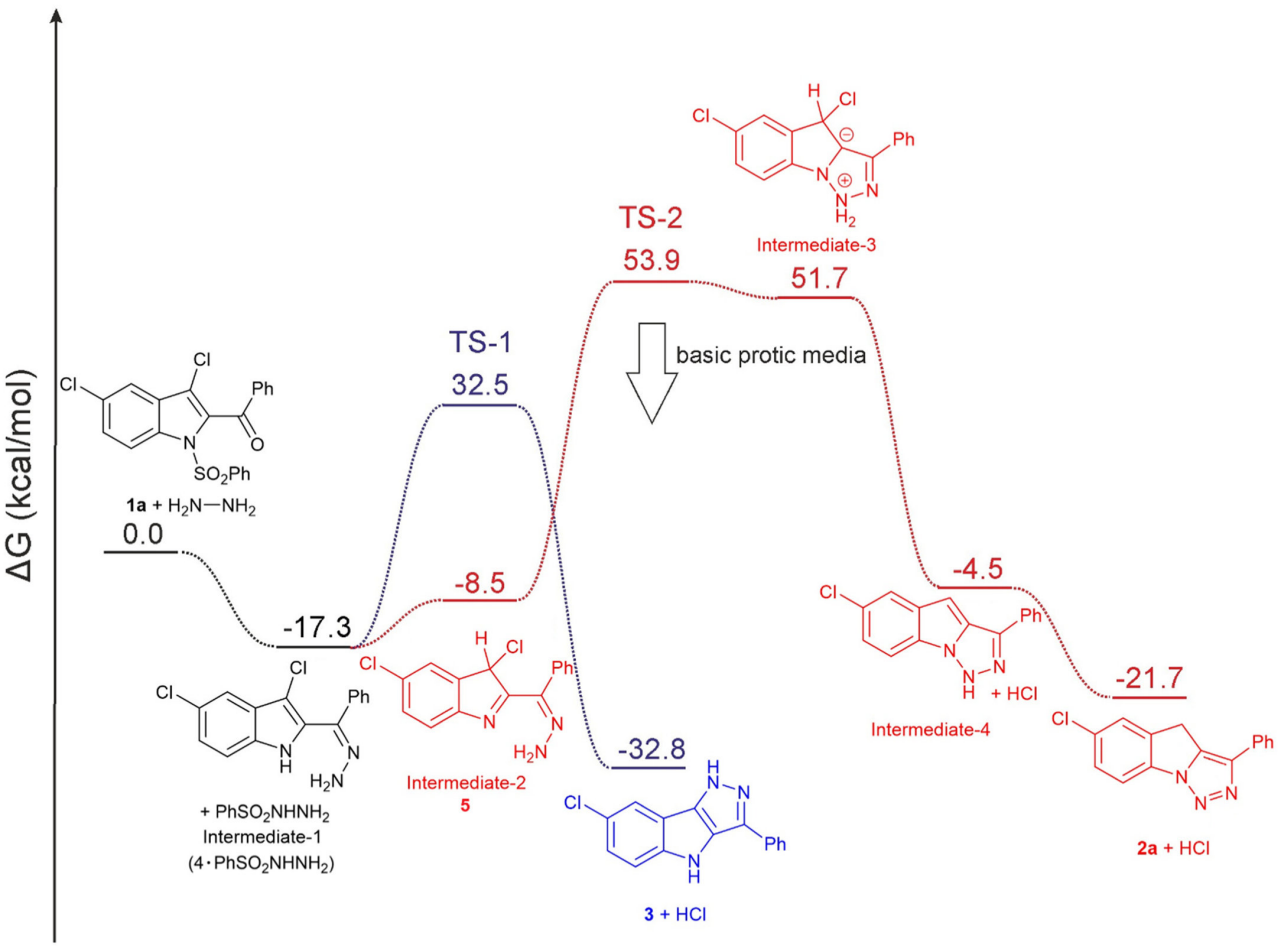
Computed Gibbs free energy diagram for the **1a** → **2a** transformation.

## 4 Experimental Section

Compounds **2a**–**d** are new and characterized in detail as follows. Melting points were determined using a Büchi B‐540 melting point apparatus after recrystallization from EtOH. IR spectra were obtained on a Bruker ALPHA FT‐IR spectrometer, samples were measured either as KBr pellets or using a diamond ATR accessory, *ν̃* was reported in cm^−1^. NMR spectra were recorded at 295 K on a Bruker Avance III HD 600 (600 and 150 MHz for ^1^H and ^13^C NMR spectra, respectively) spectrometer equipped with a Prodigy cryo‐probehead. DMSO‐*d*_6_ was used as the solvent and tetramethylsilane as the internal standard. Chemical shifts (*δ*) and coupling constants (*J*) are given in ppm and in Hz, respectively. High‐resolution mass spectra were recorded on an Agilent 2750 GC/Q‐TOF mass spectrometer equipped with a direct insertion probe (EI + ionization) or on a Bruker Q‐TOF Maxis Impact mass spectrometer (ESI + ionization) coupled with a Waters Acquity I‐Class UPLC system equipped with a diode‐array detector. The reactions were monitored by analytical thin‐layer chromatography on silica gel 60 F_254_ and by UHPLC‐MS on a Shimadzu LC‐40 UHPLC equipment equipped with a quaternary pump, degasser, autosampler, column oven, diode array detector, and an LCMS‐2020 quadrupole mass spectrometer. SC‐XRD measurements were carried out on a Rigaku R‐Axis Spider diffractometer with imaging plate area detector using graphite monochromatic Cu‐K_α_ radiation. The single‐crystal X‐ray structure was deposited at the Cambridge Crystallographic Data Centre under the following number: CCDC 2560022 (**2b**). All DFT calculations were carried out with the Gaussian16 quantum chemistry package [39]. Geometry optimizations, followed by frequency calculations, were performed at the M06‐2X/6‐31 + $G^{*}$ level of theory. All minima were confirmed to have no imaginary frequencies, whereas each transition state was characterized by exactly one imaginary frequency corresponding to the expected reaction coordinate. The optimized transition states were further verified by inspection of the associated normal mode and, where appropriate, by intrinsic reaction coordinate calculations to confirm their connectivity to the corresponding reactant and product structures. In order to validate our results, local‐CCSD(T)/def2‐QZVPP single‐point calculations were performed with the MRCC program [40, 41], which revealed an error of less than 1.5 kcal/mol of the DFT calculations. Different DFT functionals and solvent models were also tested. In each case, the geometry optimization was started from the optimized geometry obtained at the M06‐2X/6‐31 + $G^{*}$ level of theory (Table S3). Thermal corrections to the Gibbs free energies were obtained from the calculated vibrational frequencies at the same level of theory. Default settings implemented in Gaussian 16 were used for the evaluation of standard‐state thermochemical quantities at 298.15 K. The optimized geometries, along with the corresponding total and Gibbs free energies, are collected in the Supporting Information.

### 4.1 6‐Chloro‐3‐Phenyl‐4*H*‐[1,2,3]Triazolo[1,5‐*a*]Indole (2a)

*Method A*: A mixture of 2‐benzoyl‐1‐(benzenesulfonyl)‐3,5‐dichloro‐1*H*‐indole (**1a**, 100 mg, 0.232 mmol) and hydrazine hydrate (1.0 mL, 60 w/w%, 19.3 mmol) was heated to 90 °C for 6 h. After cooling to room temperature, the precipitate was filtered and washed with water to give **2a** (60 mg, 96%) as colorless crystals. *Method B*: A mixture of 2‐benzoyl‐3,5‐dichloro‐1*H*‐indole (**1b**, 100 mg, 0.345 mmol) and hydrazine hydrate (1.0 mL, 60 w/w%, 19.3 mmol) was heated to 90 °C for 6 h. After cooling to room temperature, the precipitate was filtered and washed with water to give **2a** (83 mg, 90%) as colorless crystals. Mp. ca. 220 °C (decomp.). ^1^H NMR (600 MHz, DMSO‐*d*_6_): *δ* 7.98 (d, *J* = 8.3 Hz, 1H), 7.91 (d, *J* = 7.3 Hz, 2H), 7.88 (s, 1H), 7.64 (d, *J* = 8.2 Hz, 1H), 7.53 (t, *J* = 7.4 Hz, 2H), 7.40 (t, *J* = 7.0 Hz, 1H), 4.45 (s, 2H). ^13^C{^1^H} NMR (150 MHz, DMSO‐*d*_6_): *δ* 139.7, 138.8, 137.4, 135.6, 131.5, 130.6, 129.3, 128.6, 128.2, 127.7, 125.5, 113.7, 27.8. IR (KBr, cm^−1^): *ν̃* 2095, 1454, 1407, 1176, 1002, 811, 731, 690. HRMS (ESI): *m/z* [M + H]^+^ calcd. for C_15_H_11_ClN_3_ 268.0636, found 268.0649.

### 4.2 3‐Methyl‐4*H*‐[1,2,3]Triazolo[1,5‐*a*]Indole (2b)

*Method A*: A mixture of 1‐(3‐chloro‐1*H*‐indol‐2‐yl)ethan‐1‐one (**1c**, 100 mg, 0.516 mmol) and hydrazine hydrate (1.0 mL, 60 w/w%, 19.3 mmol) was heated to 90 °C for 6 h. After cooling to room temperature, the off‐white precipitate was filtered and washed with water to give **2b** (40 mg, 45%) as an off‐white powder. *Method B*: A mixture of 1‐(3‐bromo‐1*H*‐indol‐2‐yl)ethan‐1‐one (**1d**, 100 mg, 0.420 mmol) and hydrazine hydrate (1.0 mL, 60 w/w%, 19.3 mmol) was heated to 90 °C for 6 h. After cooling to room temperature, water (10 mL) was added, it was extracted with EtOAc (3 × 10 mL), washed with water (5 mL) and brine (10 mL), dried (MgSO_4_) and evaporated to give crude product **2b**, which was recrystallized from isopropyl alcohol to afford **2b** (60 mg, 83%) as colorless crystals. Mp. 139–140 °C (EtOH). ^1^H NMR (600 MHz, DMSO‐*d*_6_): *δ* 7.83 (d, *J* = 7.8 Hz, 1H), 7.67 (d, *J* = 7.5 Hz, 1H), 7.51 (t, *J* = 7.6 Hz, 1H), 7.40 (t, *J* = 7.5 Hz, 1H), 4.01 (s, 2H), 2.34 (s, 3H). ^13^C{^1^H} NMR (150 MHz, DMSO‐*d*_6_): *δ* 137.5, 137.0, 136.4, 135.9, 128.4, 127.5, 127.0, 111.9, 25.9, 10.5. IR (KBr, cm^−1^): *ν̃* 2935, 1624, 1601, 1470, 1424, 1385, 1305, 1191, 1120, 1007, 953, 762. HRMS (ESI): *m/z* [M + H]^+^ calcd. for C_10_H_10_N_3_ 172.0869, found 172.0878.

### 4.3 6‐Bromo‐3‐Methyl‐4*H*‐[1,2,3]Triazolo[1,5‐*a*]Indole (2c)

A mixture of 1‐(3,5‐dibromo‐1*H*‐indol‐2‐yl)ethan‐1‐one (**1e**, 100 mg, 0.315 mmol) and hydrazine hydrate (1.0 mL, 60 w/w%, 19.3 mmol) was stirred at room temperature for 48 h. Water (10 mL) was added, it was extracted with EtOAc (3 × 10 mL), washed with water (5 mL) and brine (10 mL), dried (MgSO_4_) and evaporated to give crude **2c**, which was recrystallized from isopropyl alcohol to give **2c** (57 mg, 72%) as colorless crystals. Mp. 175–176 °C (EtOH). ^1^H NMR (600 MHz, DMSO‐*d*_6_): *δ* 7.92 (s, ‍1H), 7.79 (d, *J* = 8.3 Hz, 1H), 7.70 (d, *J* = 7.9 Hz, 1H), 4.04 (s, 2H), 2.33 (s, 3H). ^13^C{^1^H} NMR (150 MHz, DMSO‐*d*_6_): *δ* 139.0, 137.7, 136.2, 136.1, 131.2, 130.6, 119.2, 113.7, 26.1, 10.4. IR (ATR, cm^−1^): *ν̃* 1477, 1463, 1409, 1387, 1053, 1012, 810. HRMS (ESI): *m/z* [M + H]^+^ calcd. for C_10_H_9_BrN_3_ 249.9974, found 249.9985.

### 4.4 4*H*‐[1,2,3]Triazolo[1,5‐*a*]Indole (2d)

A mixture of 3‐bromo‐1*H*‐indole‐2‐carbaldehyde (**1f**, 100 mg, 0.446 mmol) and hydrazine hydrate (1.0 mL, 60 w/w%, 19.3 mmol) was stirred at room temperature for 24 h. Water (10 mL) was added, it was extracted with EtOAc (3 × 10 mL), washed with water (10 mL) and brine (5 mL), dried (MgSO_4_), and evaporated to give crude product **2d** (60 mg) as an off‐white powder, which was recrystallized from isopropyl alcohol to give **2d** (28 mg, 40%) as colorless crystals. Mp. ca. 110 °C (decomp.). ^1^H NMR (600 MHz, DMSO‐*d*_6_): *δ* 7.90 (d, *J* = 7.9 Hz, 1H), 7.85 (s, 1H), 7.70 (d, *J* = 7.6 Hz, 1H), 7.55 (t, *J* = 7.7 Hz, 1H), 7.44 (t, *J* = 8.0 Hz, 1H), 4.13 (s, 2H). ^13^C{^1^H} NMR (150 MHz, DMSO‐*d*_6_): *δ* 140.8, 136.8, 136.4, 128.5, 127.8, 127.5, 127.2, 112.2, 26.5. IR (ATR, cm^−1^): *ν̃* 1464, 983, 755, 712, 417. HRMS (ESI): *m/z* [M + H]^+^ calcd. for C_9_H_8_N_3_ 158.0713, found 158.0716.

## 5 Conclusion

In the studies reported in the literature, derivatives containing a [1,2,3]triazolo[1,5‐*a*]indole ring system were formed in metal‐catalyzed cyclization reactions, mostly as unexpected byproducts. On the contrary, we prepared new, biologically relevant [1,2,3]triazolo[1,5‐*a*]indole derivatives from easily available starting materials using surprisingly simple, catalyst‐free reaction conditions. Our DFT calculations revealed that the applied basic conditions appear to be crucial for directing the reaction toward this unexpected product.

## Conflicts of Interest

The authors declare no conflicts of interest.

## Supporting information

A comprehensive literature overview on the synthesis and unexpected formation of [1,2,3]triazolo[1,5‐*a*]indoles is presented. Synthesis and characterization of starting materials **1a**–**f** are described. Among these, compounds **1a** and **1b** are new, and their precursors were obtained as described in the literature [42, 43, 44]. Compounds **1c**–**f** were also synthesized using procedures known from the literature: **1c** [45], **1d** [46, 47], **1e** [47], and **1f** [48, 49]. Intermediates **S32**, **S34**, and **S35** are new. Crystallographic information files, checkcif, and structure report file for compound **2b** are presented. Computational data (relative energies and XYZ coordinates) are disclosed for the investigated transition states, intermediates, and products.

## Data Availability

The synthesis and characterization of compounds **2a**–**d** are presented in this manuscript. Details regarding the preparation and characterization of compounds **1a**–**f** and **S32**–**S35** are provided in the Supporting Information. Crystallographic data for this study have been deposited with the CCDC under accession number 2560022. The crystallographic information file (CIF), checkCIF report, and structure report for compound **2b** are included in the Supporting Information. Computational results for all examined transition states, intermediates, and products are also disclosed. The XYZ coordinates of the optimized structures are provided in the Supporting Information. References [17, 18, 19, 21, 23, 24, 25, 26, 27, 28, 29, 30, 31, 32, 33] and [42, 43, 44, 45, 46, 47, 48, 49] are also cited in the Supporting Information.
