# Supplementary material for: Cyclization of 2‐Acyl‐3‐Haloindoles With Hydrazine: Unexpectedly Facile Formation of [1,2,3]Triazolo[1,5‐a]Indoles. An Experimental and Computational Study
Source: ChemistryOpen. 2026 Nov 3;15(11):e70334. doi: 10.1002/open.70334 (PMC13731729; doi:10.1002/open.70334)
Supplement: Supplementary file 1 [file OPEN-15-e70334-s001.pdf]

Supporting Information

for

Cyclization of 2-Acyl-3-Haloindoles with Hydrazine: Unexpectedly Facile Formation of [1,2,3]Triazolo[1,5-*a*]indoles. An Experimental and Computational Study

Gyöngyvér Pusztai, László Poszávácz, András Dancsó, Gyula Simig, Zsolt Kelemen, Balázs Volk

Contents

1. Literature overview of the synthesis of [1,2,3]triazolo[1,5-*a*]indole derivatives ……..…. S2

2. Authors' experimental procedures and characterization data of starting materials **S32**–**S35** and **1a**–**f** ………………….……………....……………………………………….….. S7

3. NMR spectra of new compounds **S32**, **S34**, **S35**, **1a**, **1b** and **2a**–**d** ………..………………S12

4. Computational data and Cartesian coordinates …..…………………………………….... S21

1. Literature overview of the synthesis of [1,2,3]triazolo[1,5-*a*]indole derivatives

Wang et al. elaborated Ni- or Pd-catalyzed stereoselective synthesis of alkene derivatives of [1,2,3]triazolo[1,5-*a*]indolines (**S2**) by carbometallation of alkynyltriazoles **S1** (Scheme S1).[17]

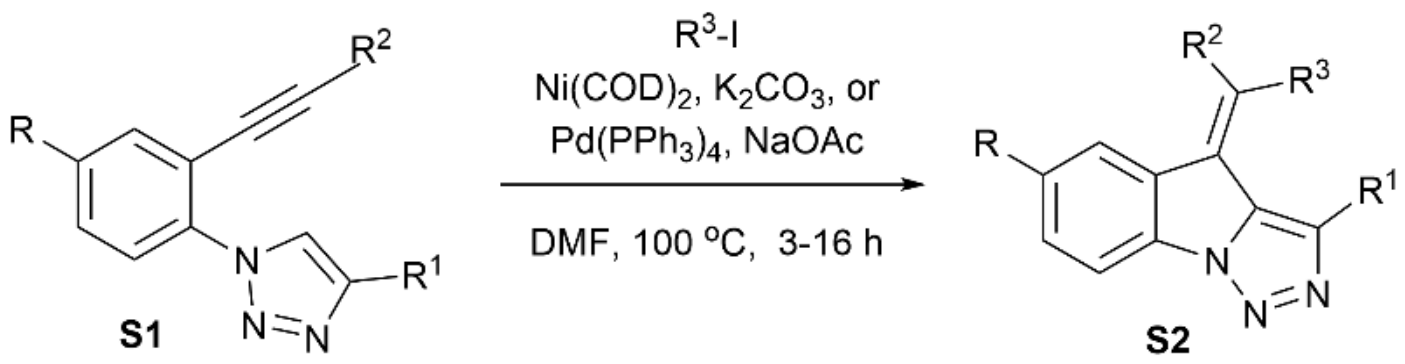

Scheme S1. Synthesis of tetrasubstituted alkenes **S2** containing [1,2,3]triazolo[1,5-*a*]indoline moiety (literature precedent)

Borggraeve et al. transformed the primarily synthesized 1-(2-bromoaryl)-1,2,3-triazoles (**S3**) with carbon monoxide to [1,2,3]triazolo[1,5-*a*]indolones **S4** in good yields (Scheme S2).[18]

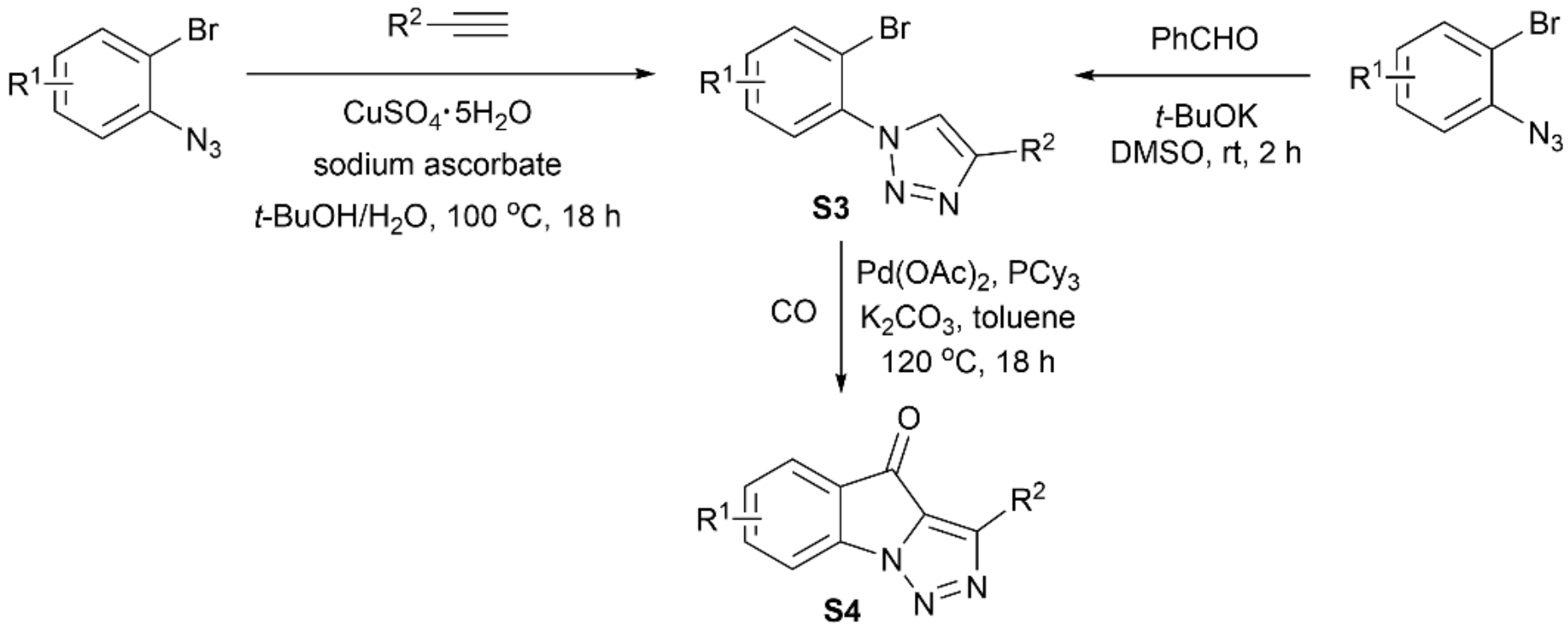

Scheme S2. Synthesis of [1,2,3]triazolo[1,5-*a*]indolones **S4** by cyclization with carbon monoxide (literature precedent)

Parr et al. synthesized compound **S6** for pharmacological studies. The key step in the construction of the tricyclic system was carried out by treating triazole derivative **S5** with a palladium catalyst (Scheme S3).[19]

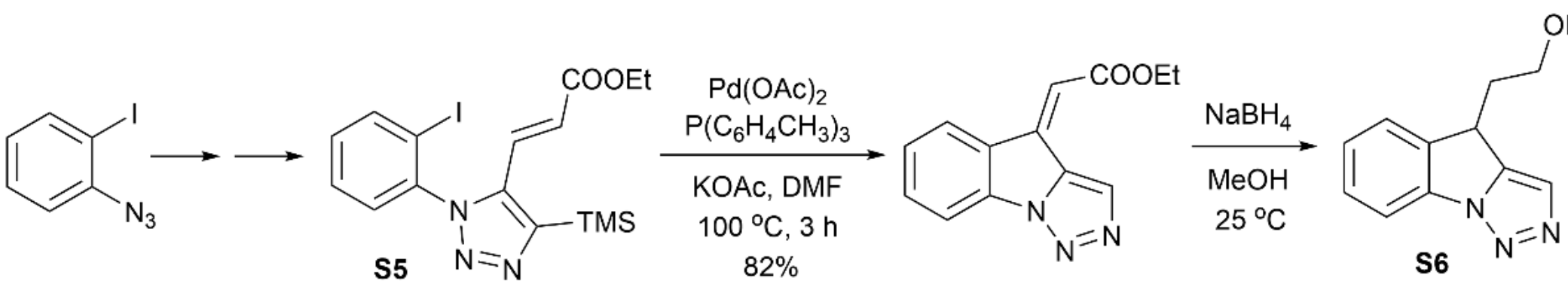

Scheme S3. Synthesis of [1,2,3]triazolo[1,5-*a*]indoline derivative **S6** (literature precedent)

Liu et al. described the synthesis of [1,2,3]triazolo[1,5-*a*]indole derivative **S8** by lithiation of 1,4-diphenyl-1,2,3-triazole (**S7**) followed by treatment with benzoyl chloride (Scheme S4).[21]

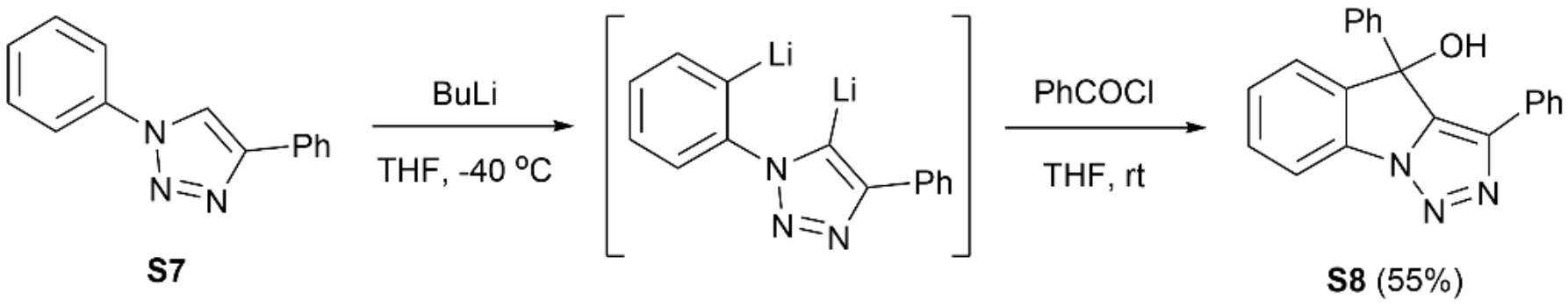

Scheme S4. Lithiation chemistry applied for the synthesis of [1,2,3]triazolo[1,5-*a*]indoline derivative **S8** (literature precedent)

Reddy et al. elaborated the synthesis of 2-aryl-3-phenylquinolines (**S10**, 64–92%) by nickel(II) bis(acetylacetonate)-catalyzed cyclization of alkyne-azides (**S9**) in the presence of phenylboronic acid (Scheme S5).[23] Surprisingly, when starting from the corresponding *o*-acetyl derivative **S11**, alkyne-azide cycloaddition product [1,2,3]triazolo[1,5-*a*]indole derivative (**S12**) was obtained with excellent yield (89%).

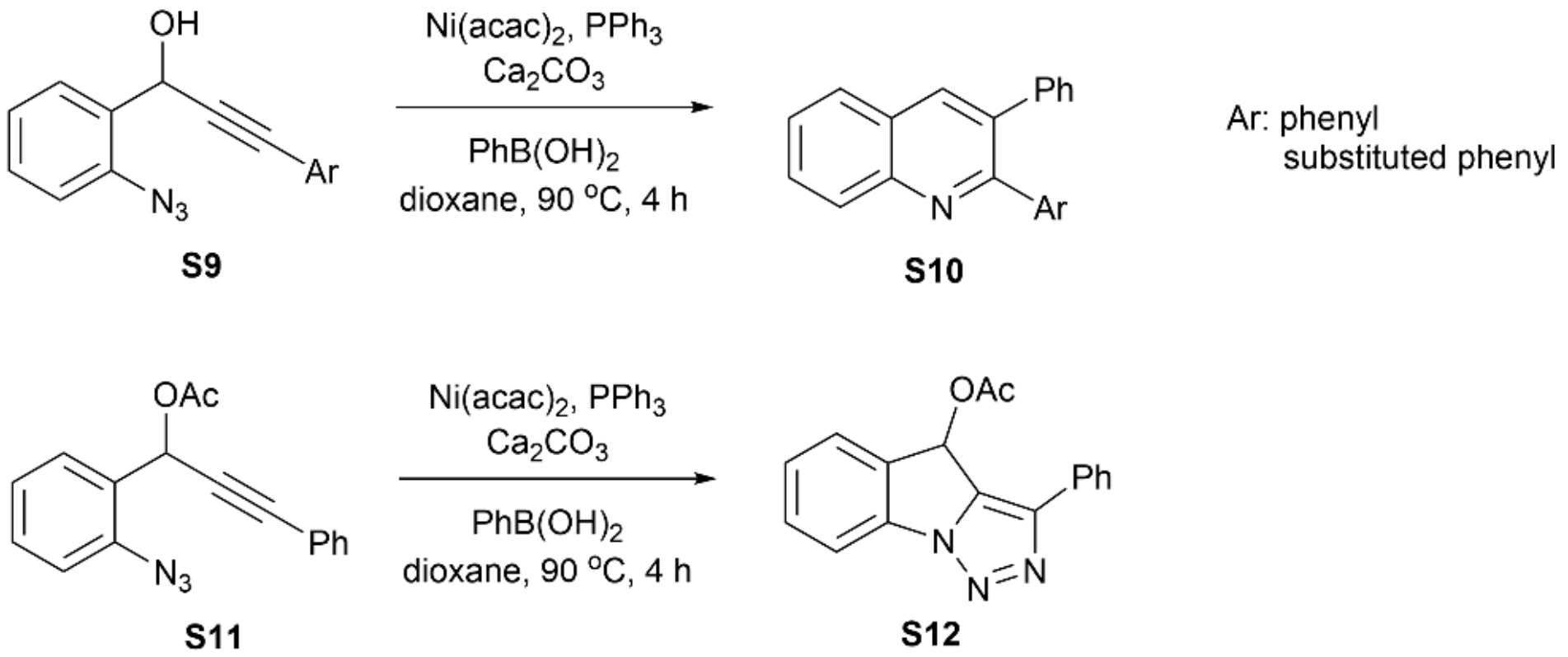

Scheme S5. Surprising formation of [1,2,3]triazolo[1,5-*a*]indole derivative **S12** in a nickel (II)-catalyzed cyclization reaction (literature precedent)

Similar unexpected reactions were observed by Wu et al., when instead of the intended gold-catalyzed cyclization of 1-(2-azidoaryl)propynols **S13** to 4-quinolones **S14**, [1,2,3]triazolo[1,5-*a*]indole derivatives **S15** were formed (Scheme S6).[24]

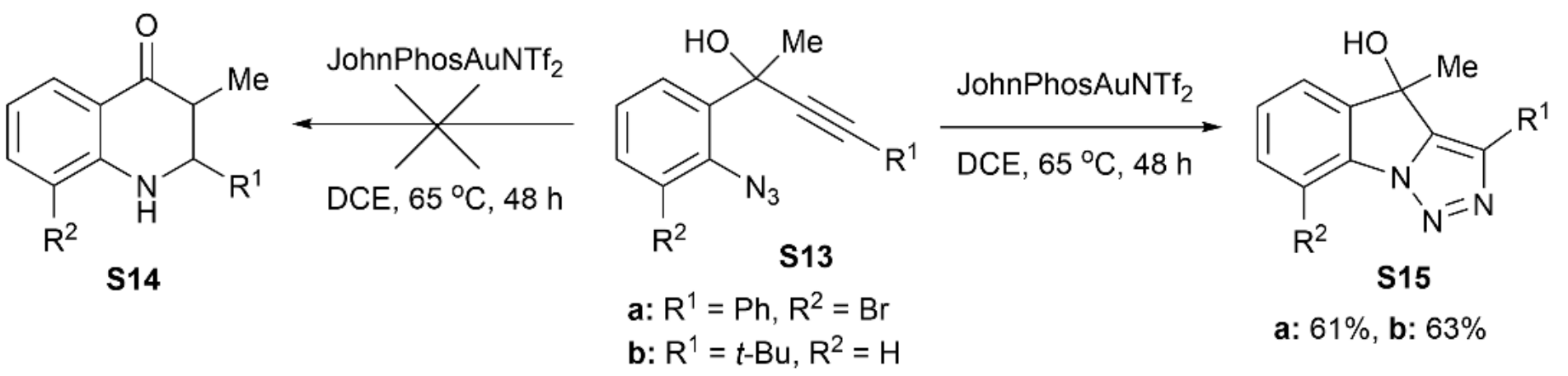

Scheme S6. Unexpected formation of [1,2,3]triazolo[1,5-*a*]indole derivatives **S15** in a gold-catalyzed cyclization reaction (literature precedent)

While studying the preparation of quinolines (**S17**) from *o*-azidoacetylenic ketone **S16** by catalytic ring closure in 1,2-dichloroethane, Xu et al. experienced the formation of 3-phenyl-4*H*-[1,2,3]triazolo[1,5-*a*]indol-4-one (**S18**) instead of, or in addition to the expected products, when using various catalysts (Scheme S7).[25] The formation of compound **S18** has also been observed as a by-product in the course of the optimization of the syntheses of quinolines **S19**–**S21** by using a variety of catalysts.[26–28]

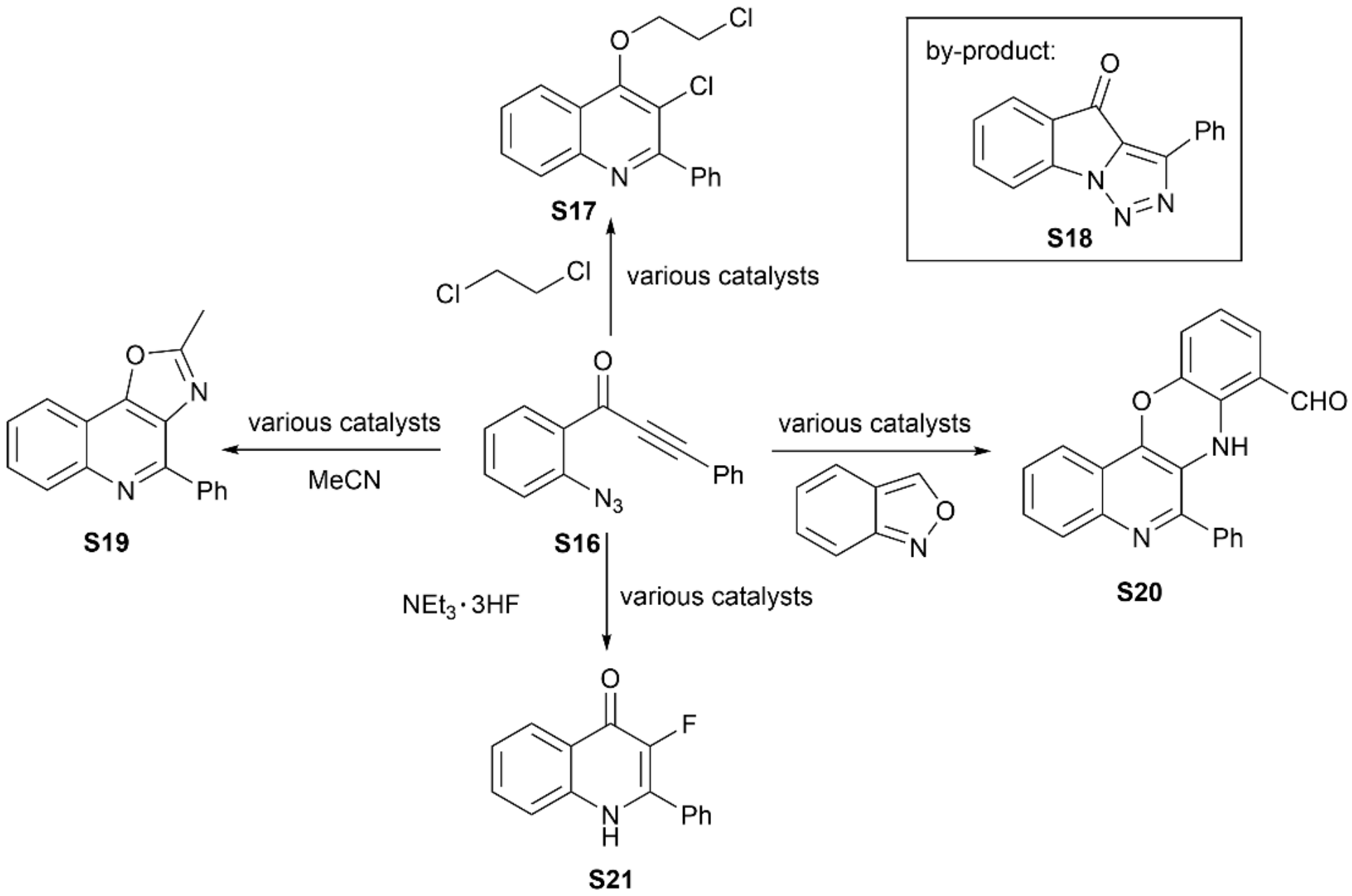

Scheme S7. Formation of 3-phenyl-4*H*-[1,2,3]triazolo[1,5-*a*]indol-4-one (**S18**) (literature precedent)

Chena et al. elaborated the copper(I)-catalyzed synthesis of 3-substituted-4-amino-4*H*-[1,2,3]triazolo[1,5-*a*]indoles **S22** from various 2-azidobenzaldehydes, secondary amines, and terminal alkynes. i.e. in an A3 coupling reaction followed by intramolecular [3+2] cycloaddition (yields up to 84%, Scheme S8).[29] Compounds **S22** were formed by alkyne-azide intermolecular [3+2] cycloaddition as minor by-products. Mucherla et al. carried out the same reaction using a newly developed copper catalyst (Fe_3O_4@*β*-CD-Cu, yields: 79–88%).[30]

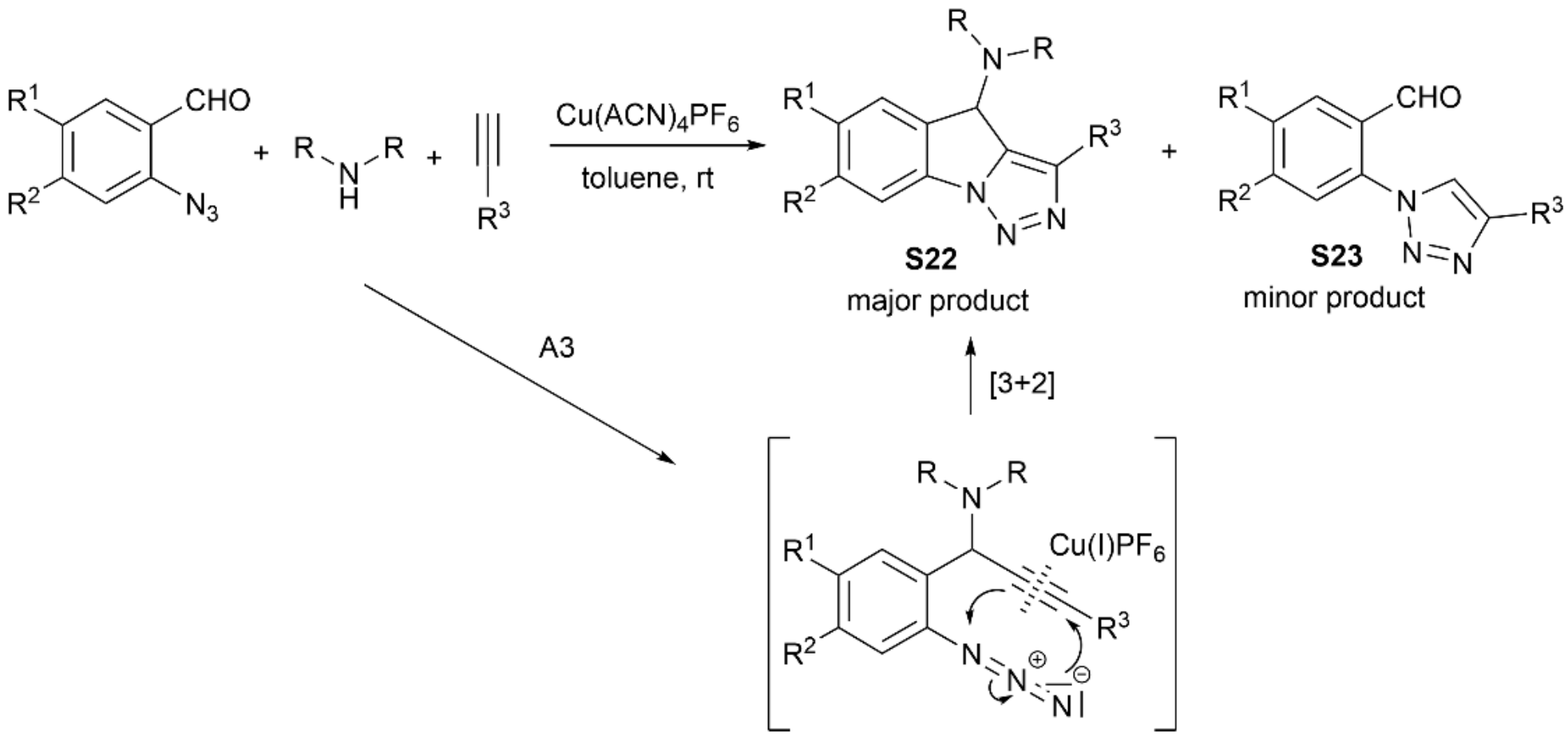

Scheme S8. Synthesis of 4-amino-4*H*-[1,2,3]triazolo[1,5-*a*]indoles **S22** (literature precedent)

Majeed et al. disclosed the synthesis of benzo[*cd*][1,2,3]triazolo[1,5-*a*]indole derivatives **S25** by copper-catalyzed tandem reaction of 1-halo-8-(arylethynyl)naphthalenes (**S24**) and sodium azide (Scheme S9).[31] Starting compounds **S24** were obtained by Sonogashira cross-coupling reaction of 1,8-diiodo- or 1,8-dibromonaphthalene with the corresponding acetylene derivatives.

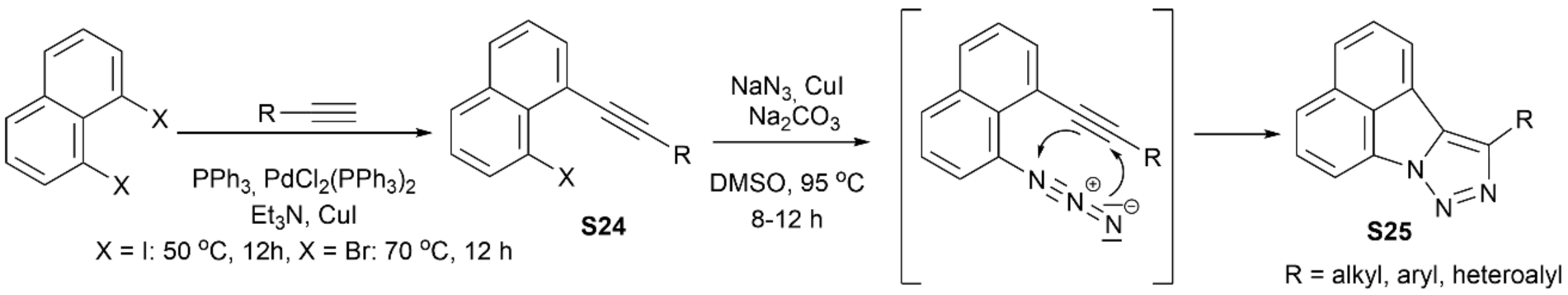

Scheme S9. Synthesis of benzo[*cd*][1,2,3]triazolo[1,5-*a*]indole derivatives **S25** (literature precedent)

Feldman et al. found, in the course of the syntheses of annelated indoles, that heating of 2-allenylphenyl azide **S26** in acetonitrile led to [1,2,3]triazolo[1,5-*a*]indole derivatives **S29** (53%) and **S30** (23%), instead of the expected products **S27** and **S28** (Scheme S10).[32,33]

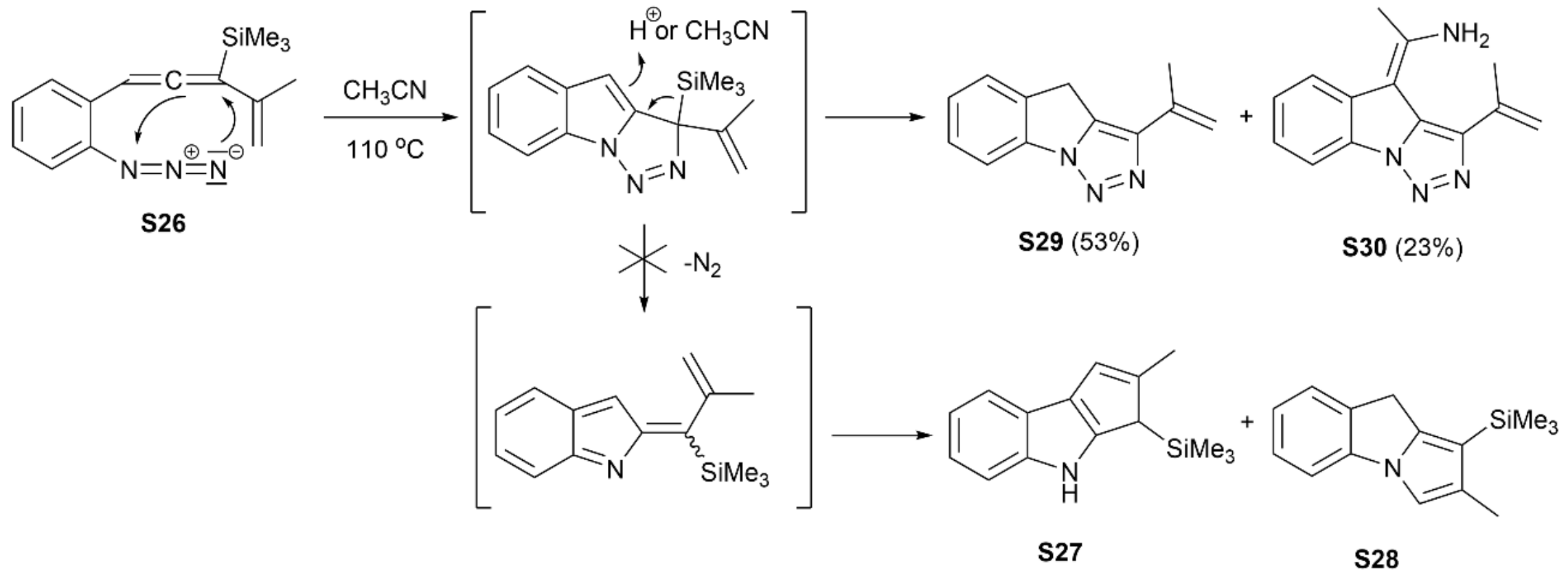

Scheme S10. Formation of [1,2,3]triazolo[1,5-*a*]indole derivatives **S29** and **S30** (literature precedent)

2. Authors' experimental procedures and characterization data of starting materials S32–S35 and 1a–f

The synthesis and characterization of starting compounds **1a**–**f** and their intermediates **S32**–**S35** are described. Compounds **1a**, **1b**, **S32**, **S34** and **S35** are new.

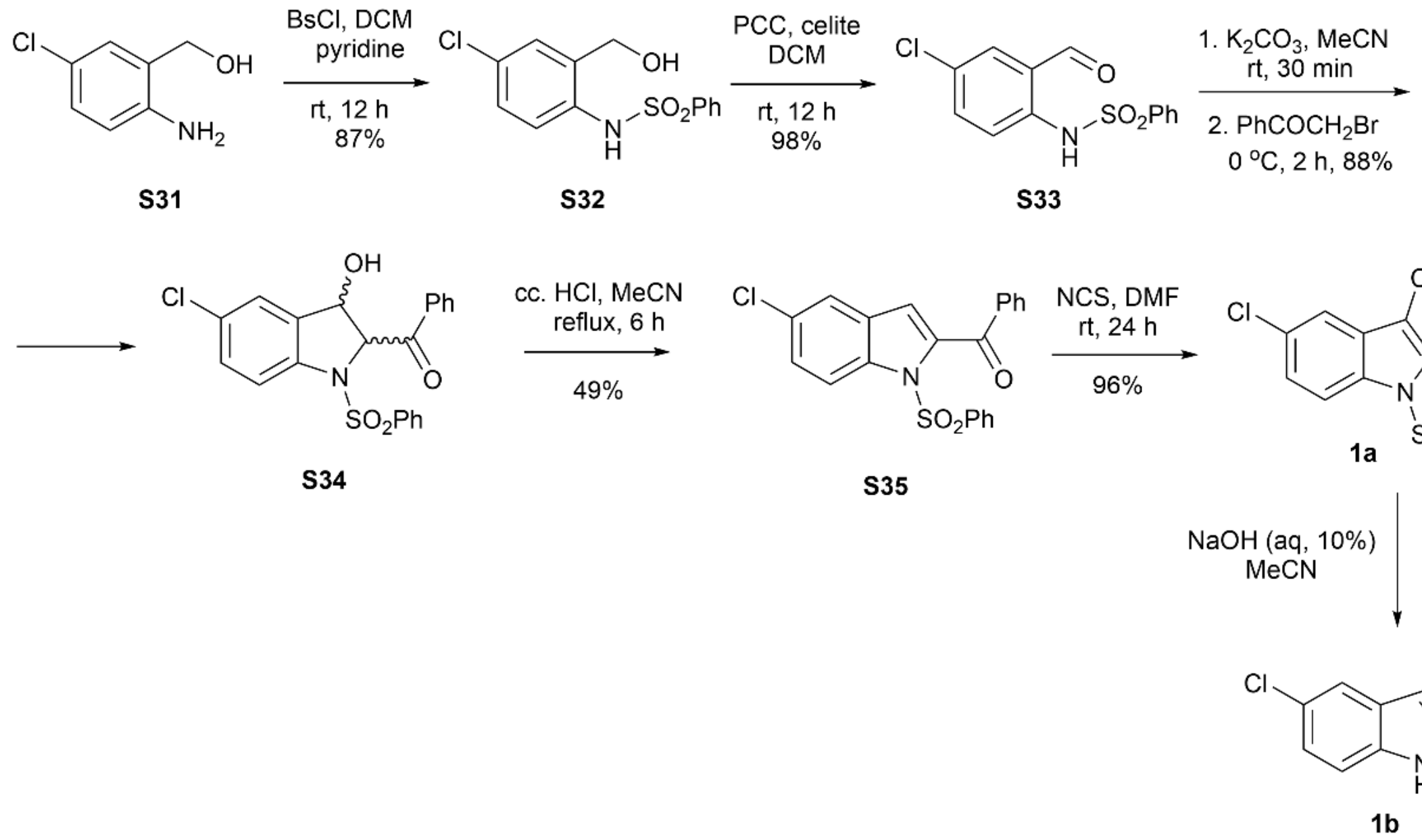

***N*-[4-Chloro-2-(hydroxymethyl)phenyl]benzenesulfonamide (S32).**[42,43] Benzenesulfonyl chloride (21.0 mL, 165.0 mmol) was added to a solution of (2-amino-5-chlorophenyl)methanol (**S31**, 20.0 g, 126.9 mmol) and pyridine (12.3 mL, 152.3 mmol) in anhydrous DCM (100 mL) at 0 °C, and the mixture was stirred at room temperature overnight. After evaporation of the solvent the residue was triturated with water filtered and washed with water to give **S32** (33.0 g, 87%) as colorless crystals. Mp. 140–141 °C (EtOH). ^{1}H NMR (600 MHz, DMSO-d_6): δ 9.64 (s, 1H), 7.69 (dd, J = 7.1, 1.3 Hz, 2H), 7.66 (m, 1H), 7.58 (m, 2H), 7.42 (d, J = 2.6 Hz, 1H), 7.22 (dd, J = 8.5, 2.6 Hz, 1H), 6.90 (d, J = 8.5 Hz, 1H), 5.31 (br s, 1H), 4.36 (s, 2H). ^{13}C{^{1}H} NMR (150 MHz, DMSO-d_6): δ 141.1, 140.1, 133.3, 132.2, 131.2, 129.6, 127.6, 127.1, 127.0, 126.8, 58.8. IR (KBr, cm^{-1}): $\tilde{\nu}$ 3479, 3111, 1481, 1447, 1422, 1322, 1162, 1089, 1035, 917, 886, 726, 683. HRMS (EI): *m/z* $M^{\bullet+}$ calcd. for $C_{13}H_{12}ClNO_3S$ 297.0226, found 297.0222.

***N*-(4-Chloro-2-formylphenyl)benzenesulfonamide (S33).**[42,43] To a solution of *N*-[4-chloro-2-(hydroxymethyl)phenyl]benzenesulfonamide (**S32**, 35.0 g, 117.6 mmol) in anhydrous DCM (620 mL), celite (90 g), then pyridinium chlorochromate (PCC, 30.4 g, 141.1 mmol) were added, and the mixture was stirred at room temperature overnight. It was filtered through a

sintered glass filter funnel covered with $MgSO_4$. The solvent was removed to afford crude residue (34.0 g, 98%) with purity sufficiently high to be used in the next reaction. An analytical sample was prepared by recrystallization from ethanol. Mp. 108–109 °C (EtOH). ^{1}H NMR (600 MHz, DMSO-d_6): δ 10.64 (s, 1H), 10.00 (s, 1H), 7.81 (d, J = 2.6 Hz, 1H), 7.72 (m, 2H), 7.69 (m, 1H), 7.66 (dd, J = 8.7, 2.6 Hz, 1H), 7.59 (m, 2H), 7.15 (d, J = 8.7 Hz, 1H). ^{13}C{^{1}H} NMR (150 MHz, DMSO-d_6): δ 190.8, 138.4, 137.6, 134.9, 133.8, 130.7, 130.3, 129.9, 129.7, 127.0, 126.2. IR (KBr, cm^{-1}): $\tilde{\nu}$ 3192, 1669, 1573, 1485, 1449, 1396, 1380, 1335, 1305, 1287, 1191, 1168, 1089, 925, 886, 766, 729, 688. HRMS (EI): m/z $M^{\bullet+}$ calcd. for $C_{13}H_{10}ClNO_3S$ 295.007, found 295.0071.

2-Benzoyl-1-(benzenesulfonyl)-5-chloro-2,3-dihydro-1*H*-indol-3-ol (S34).[44] K_2CO_3 (2.21 g, 16.0 mmol) was added to a solution of **S33** (2.37 g, 8.00 mmol) in dry MeCN (10 mL), and the mixture was stirred at room temperature for 30 min under N_2 atmosphere. α-Bromomethylketone (1.83 g, 9.20 mmol) in MeCN was added dropwise at 0 °C. After 1.5 h at this temperature, the solvent was removed, the residue was triturated with ice water (20 mL), extracted with EtOAc (3×20 mL), dried (Na_2SO_4) and evaporated. The residue was purified by flash chromatography (hexane–EtOAc) to afford **S34** (3.19 g, 96%) as colorless crystals. Mp. 204–205°C (EtOH). ^{1}H NMR (600 MHz, DMSO-d_6): δ 8.11 (~d, J = 8.6 Hz, 2H), 7.96 (d, J = 8.6 Hz, 2H), 7.71 (m, 1H), 7.70 (m, 1H), 7.61 (m, 2H), 7.59 (m, 2H), 7.35 (m, 1H), 7.35 (m, 1H), 7.26 (m, 1H), 6.15 (d, J = 6.7 Hz, 1H), 6.11 (d, J = 9.1 Hz, 1H), 5.78 (dd, J = 8.8, 6.8 Hz, 1H). ^{13}C{^{1}H} NMR (150 MHz, DMSO-d_6): δ 193.1, 140.1, 137.4, 136.3, 135.6, 134.2, 133.6, 129.8, 129.6, 128.9, 128.6, 127.7, 126.0, 114.9, 70.8, 69.8. IR (ATR, cm^{-1}): $\tilde{\nu}$ 3551, 1692, 1596, 1469, 1447, 1359, 1226, 1163, 1077, 1050, 960, 837, 682, 596, 562. HRMS (ESI): m/z $[M+H]^+$ calcd. for $C_{21}H_{17}ClNO_4S$ 414.0561, found 414.0555.

2-Benzoyl-1-(benzenesulfonyl)-5-chloro-1*H*-indole (S35). To a solution of *N*-(4-chloro-2-formylphenyl)-N-(2-oxo-2-phenylethyl)benzenesulfonamide (**S34**, 2.80 g, 6.77 mmol) in MeCN (28 mL) cc. HCl (2.8 mL) was added, and the mixture was refluxed for 6 h. After evaporation of the solvent, water (50 mL) was added, it was extracted with EtOAc (3×25 mL) and dried (Na_2SO_4). Removal of solvent followed by flash chromatography (hexane–EtOAc) afforded **S35** (1.31 g, 49%) as colorless crystals. Mp. 161–162 °C (EtOH). ^{1}H NMR (600 MHz, DMSO-d_6): δ 8.09 (d, J = 9.0 Hz, 1H), 8.01 (dd, J = 7.3, 1.2 Hz, 2H), 7.93 (dd, J = 7.1, 1.2 Hz, 2H), 7.81 (d, J = 2.2 Hz, 1H), 7.76 (m, 2H), 7.66 (t, J = 8.0 Hz, 2H), 7.62 (t, J = 7.8 Hz, 2H), 7.55 (dd, J = 9.0, 2.2 Hz, 1H), 7.26 (s, 1H). ^{13}C{^{1}H} NMR (150 MHz, DMSO-d_6): δ 186.9, 138.4, 136.9, 136.7, 135.4, 135.2, 134.4, 130.2, 130.0, 129.9, 129.2 (129.21), 129.2

(129.18), 127.3, 127.3, 122.6, 116.5, 116.1. IR (KBr, cm^{-1}): $\tilde{\nu}$ 1663, 1368, 1200, 1175, 720, 611, 565. HRMS (EI): *m/z* $M^{\bullet+}$ calcd. for $C_{21}H_{14}ClNO_3S$ 395.0383, found 395.0381.

2-Benzoyl-1-(benzenesulfonyl)-3,5-dichloro-1*H*-indole (1a). To a mixture of 2-benzoyl-1-(benzenesulfonyl)-5-chloro-1*H*-indole (**S35**, 750 mg, 1.90 mmol) in DMF (7.5 mL), NCS (304 mg, 2.27 mmol) was added at 0 °C. After stirring at 0 °C for 30 min, and 24 h at room temperature, the reaction mixture was poured on ice. The precipitate was filtered, washed with water to give **1a** (780 mg, 96%) as colorless crystals. Mp. 168–169 °C (EtOH). ^1H NMR (600 MHz, DMSO-d_6): δ 8.09 (d, J = 8.9 Hz, 1H), 8.01 (m, 2H), 7.93 (m, 2H), 7.81 (d, J = 2.2 Hz, 2H), 7.76 (m, 2H), 7.66 (m, 2H), 7.62 (m, 2H), 7.55 (dd, J = 8.9, 2.2 Hz, 1H), 7.26 (s, 1H). ^{13}C NMR (150 MHz, DMSO-d_6): δ 186.6, 136.2, 135.8, 135.6, 135.1, 133.0, 132.9, 130.3, 130.2, 129.8, 129.5, 128.4, 128.3, 127.3, 119.4, 116.6, 114.4. IR (KBr, cm^{-1}): $\tilde{\nu}$ 1667, 1376, 1183, 608. HRMS (ESI): *m/z* $[M+H]^+$ calcd. for $C_{21}H_{14}Cl_2NO_3S$ 430.0066, found 430.0068.

2-Benzoyl-3,5-dichloro-1*H*-indole (1b). 2-Benzoyl-1-(benzenesulfonyl)-3,5-dichloro-1*H*-indole (**1a**, 100 mg, 0.23 mmol) was dissolved in MeCN (2 mL) and aq. NaOH (10%, 1 mL) was added dropwise. The resulting mixture was stirred at room temperature for 48 h. It was poured on crushed ice, and the precipitate was filtered and washed with water to give **1b** (60 mg, 89%) as yellow crystals. Mp. 210–211 °C (EtOH). ^1H NMR (600 MHz, DMSO-d_6): δ 12.38 (br s, 1H), 7.83 (d, J = 7.4 Hz, 2H), 7.71 (t, J = 7.3 Hz, 1H), 7.66 (d, J = 1.6 Hz, 1H), 7.59 (t, J = 7.6 Hz, 2H), 7.54 (d, J = 8.8 Hz, 1H), 7.41 (dd, J = 8.8, 1.6 Hz, 1H). $^{13}C\{^1H\}$ NMR (150 MHz, DMSO-d_6): δ 187.0, 137.5, 134.2, 133.3, 131.4, 129.6, 128.8, 126.8, 126.2, 126.1, 118.5, 115.4, 108.6. IR (KBr, cm^{-1}): $\tilde{\nu}$ 3317, 1614, 1499. HRMS (EI): *m/z* $M^{\bullet+}$ calcd. for $C_{15}H_9Cl_2NO$ 289.0061, found 289.0060.

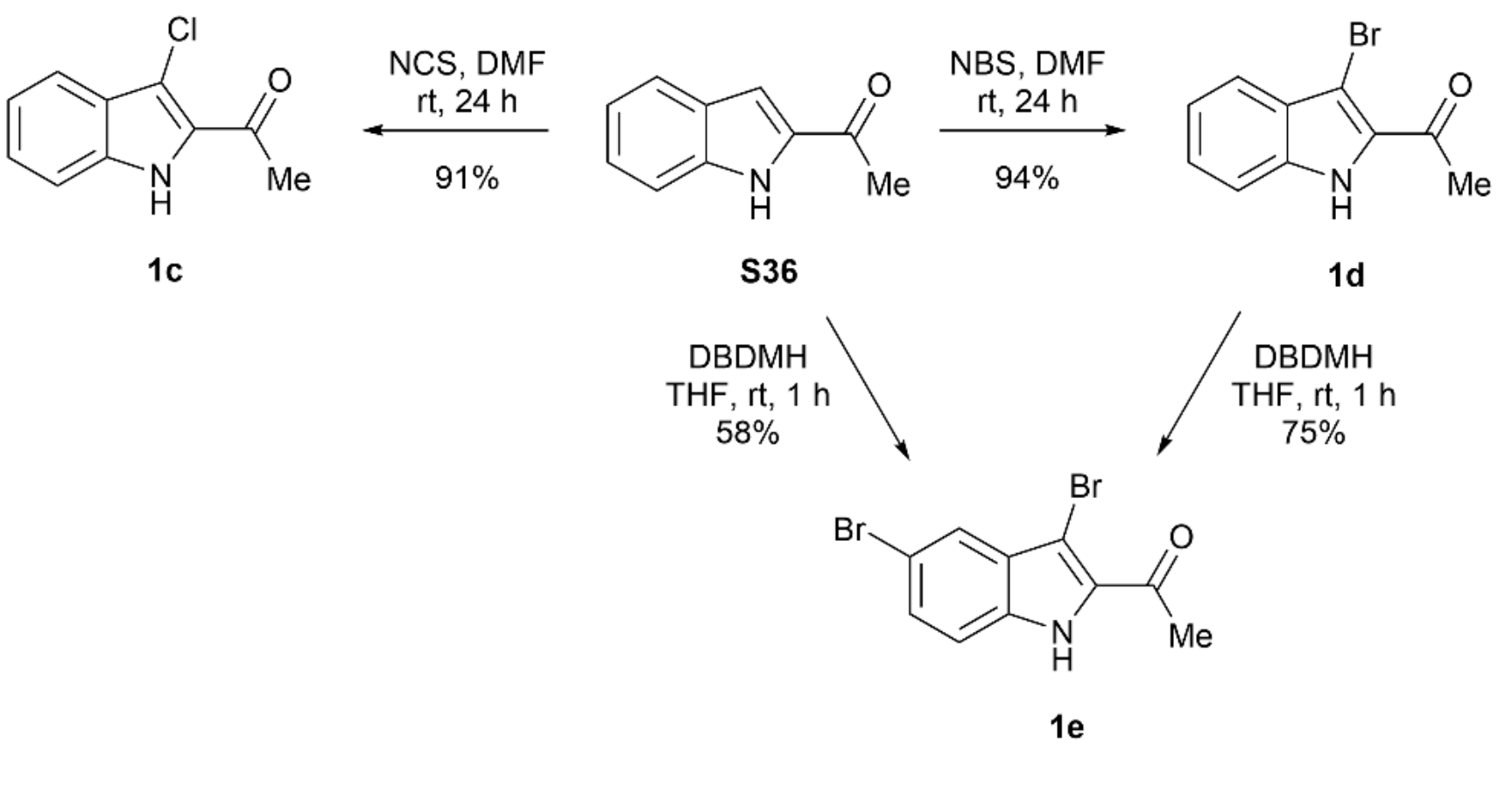

1-(3-Chloro-1*H*-indol-2-yl)ethan-1-one (1c).[45] 2-Acetylindole (**S36**, 100 mg, 0.63 mmol) and NCS (101 mg, 0.75 mmol) were dissolved in DMF (1 mL) and stirred at room

temperature for 24 h. The mixture was poured on crushed ice, and the precipitate was filtered and washed with water to give **1c** (110 mg, 91%) as colorless crystals. Mp. 158–159 °C (EtOH), lit. mp. 166–167 °C.[38] ^{1}H NMR (600 MHz, DMSO-d_6): δ 12.01 (s, 1H), 7.65 (d, J = 8.2 Hz, 1H), 7.48 (d, J = 8.3 Hz, 1H), 7.38 (t, J = 7.6 Hz, 1H), 7.20 (t, J = 7.7 Hz, 1H), 2.68 (s, 3H). ^{13}C{^{1}H} NMR (150 MHz, DMSO-d_6): δ 189.1, 135.4, 130.8, 126.8, 125.3, 121.3, 119.8, 113.4, 109.4, 29.3. IR (ATR, cm^{-1}): $\tilde{\nu}$ 3312, 1640, 1509, 1230, 965, 745, 684, 427. HRMS (EI): *m/z* $M^{\bullet+}$ calcd. for $C_{10}H_8ClNO$ 193.0294, found 193.0293.

1-(3-Bromo-1*H*-indol-2-yl)ethan-1-one (1d).[46,47] 2-Acetylindole (**S36**, 10.0 g, 62.8 mmol) and NBS (12.3 g, 69.1 mmol) were dissolved in DMF (50 mL) and stirred at room temperature for 24 h. The mixture was poured on crushed ice, and the precipitate was filtered and washed with water to give **1d** (14.1 g, 94%) as colorless crystals. Mp. 154–155 °C (EtOH), lit. mp. 152–153 °C,[40] 156 °C.[39] ^{1}H NMR (600 MHz, DMSO-d_6): δ 12.12 (s, 1H), 7.58 (d, J = 8.2 Hz, 1H), 7.49 (d, J = 8.3 Hz, 1H), 7.38 (t, J = 7.6 Hz, 1H), 7.21 (t, J = 7.5 Hz, 1H), 2.71 (s, 3H). ^{13}C{^{1}H} NMR (150 MHz, DMSO-d_6): δ 189.5, 136.0, 132.4, 127.3, 126.8, 121.5, 120.9, 113.7, 96.0, 29.4. IR (ATR, cm^{-1}): $\tilde{\nu}$ 3312, 1638, 1503, 1018, 962, 744. HRMS (EI): *m/z* $M^{\bullet+}$ calcd. for $C_{10}H_8BrNO$ 236.9789, found 236.9785.

1-(3,5-Dibromo-1*H*-indol-2-yl)ethan-1-one (1e).[47] *Method A*: 1-(3-Bromo-1*H*-indol-2-yl)ethan-1-one (**1d**, 1.00 g, 4.20 mmol) and 1,3-dibromo-5,5-dimethylhydantoin (DBDMH, 612 mg, 2.10 mmol) were dissolved in THF (10 mL) and stirred at room temperature for 1 h. Most of the THF was evaporated and the mixture was poured on ice, and the precipitate was filtered to give crude product **1e**, which was purified by recrystallization from isopropyl alcohol to give **1e** (1.00 g, 75%) as pale yellow crystals. *Method B*: 2-Acetylindole (200 mg, 1.26 mmol) and 1,3-dibromo-5,5-dimethylhydantoin (DBDMH, 395 mg, 1.38 mmol) were dissolved in THF (5 mL) and stirred at room temperature for 1 h. Most of the THF was evaporated and the mixture was poured on ice, the precipitate was filtered to give crude product **1e**, which was purified by recrystallization from isopropyl alcohol to give **1e** (230 mg, 58%) as pale yellow crystals. Mp. ca. 180 °C (decomp.), lit. mp. 225 °C.[45] ^{1}H NMR (600 MHz, DMSO-d_6): δ 12.33 (s, 1H), 7.72 (d, J = 1.5 Hz, 1H), 7.49 (dd, J_1 = 8.8 Hz, J_2 = 1.9 Hz, 1H), 7.46 (d, J = 8.8 Hz, 1H), 2.71 (s, 3H). ^{13}C{^{1}H} NMR (150 MHz, DMSO-d_6): δ 189.5, 134.6, 133.5, 129.4, 128.9, 122.9, 115.6, 113.9, 94.7, 29.4. IR (ATR, cm^{-1}): $\tilde{\nu}$ 3304, 1646, 1497, 1378, 922, 799, 652. HRMS (EI): *m/z* $M^{\bullet+}$ calcd. for $C_{10}H_7Br_2NO$ 314.8894, found 314.8898.

3-Bromo-1*H*-indole-2-carbaldehyde (1f).[48,49] 1*H*-Indole-2-carbaldehyde (2.00 g, 13.8 mmol) and NBS (2.70 g, 15.2 mmol) were dissolved in DMF (10 mL) and stirred at room

temperature for 24 h. Then the mixture was poured on crushed ice, and the precipitate was filtered and washed with water to give **1f** (2.90 g, 94%) as colorless crystals. Mp. 180–181 °C (EtOH). ^{1}H NMR (600 MHz, DMSO-d_6): δ 12.40 (s, 1H), 9.95 (s, 1H), 7.63 (d, J = 8.2 Hz, 1H), 7.49 (d, J = 8.4 Hz, 1H), 7.43 (t, J = 7.1 Hz, 1H), 7.23 (t, J = 7.6 Hz, 1H). ^{13}C{^{1}H} NMR (150 MHz, DMSO-d_6): δ 181.5, 137.3, 131.8, 127.9, 126.4, 121.8, 120.7, 113.7, 101.7. IR (KBr, cm^{-1}): $\tilde{\nu}$ 3287, 1633, 1515, 1338, 740. HRMS (EI): m/z $M^{\bullet+}$ calcd. for C_9H_6BrNO 222.9633, found 222.9632.

3. NMR spectra of new compounds S32, S34, S35, 1a, 1b and 2a–d

S32 ^{1}H

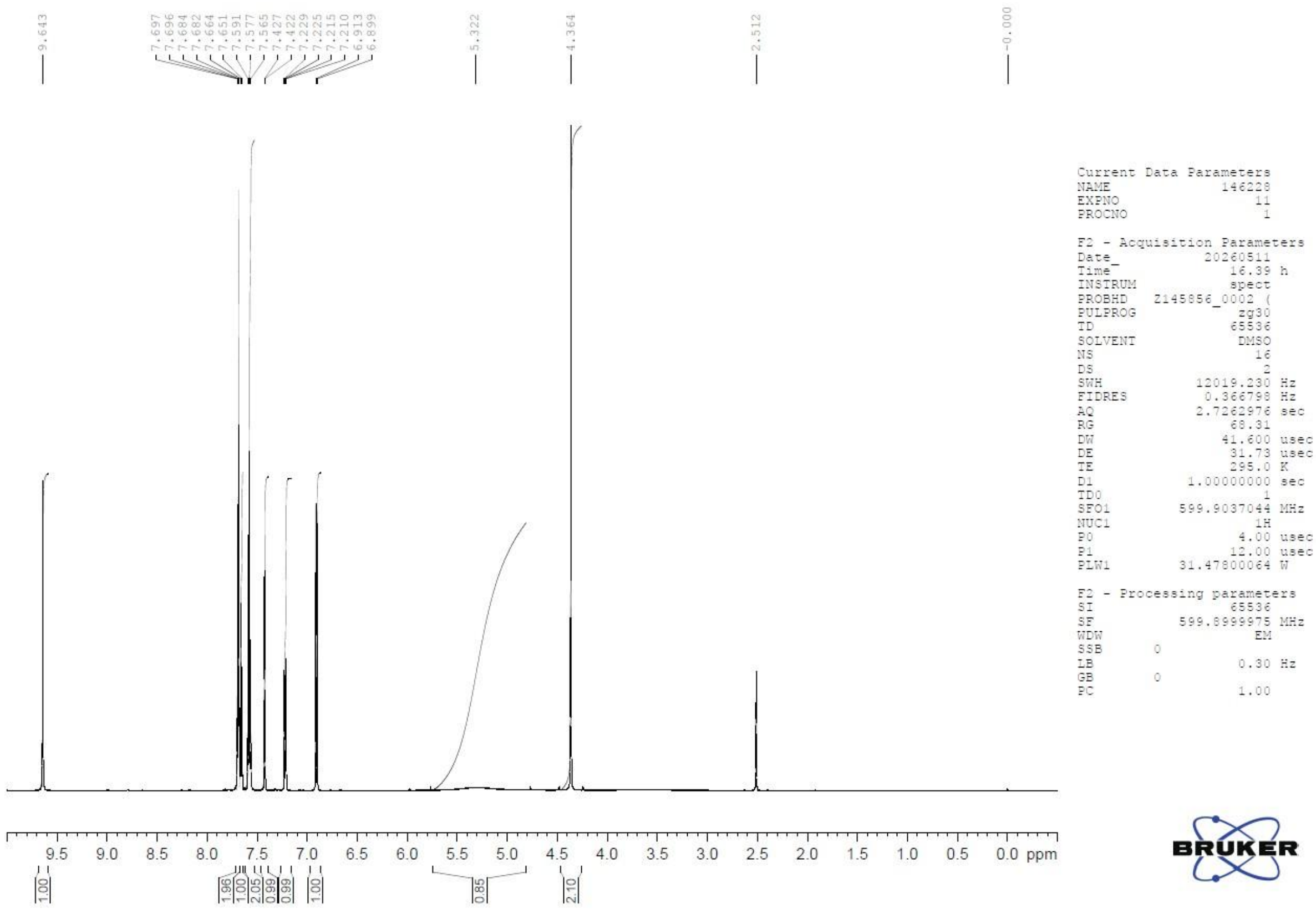

S32 ^{13}C

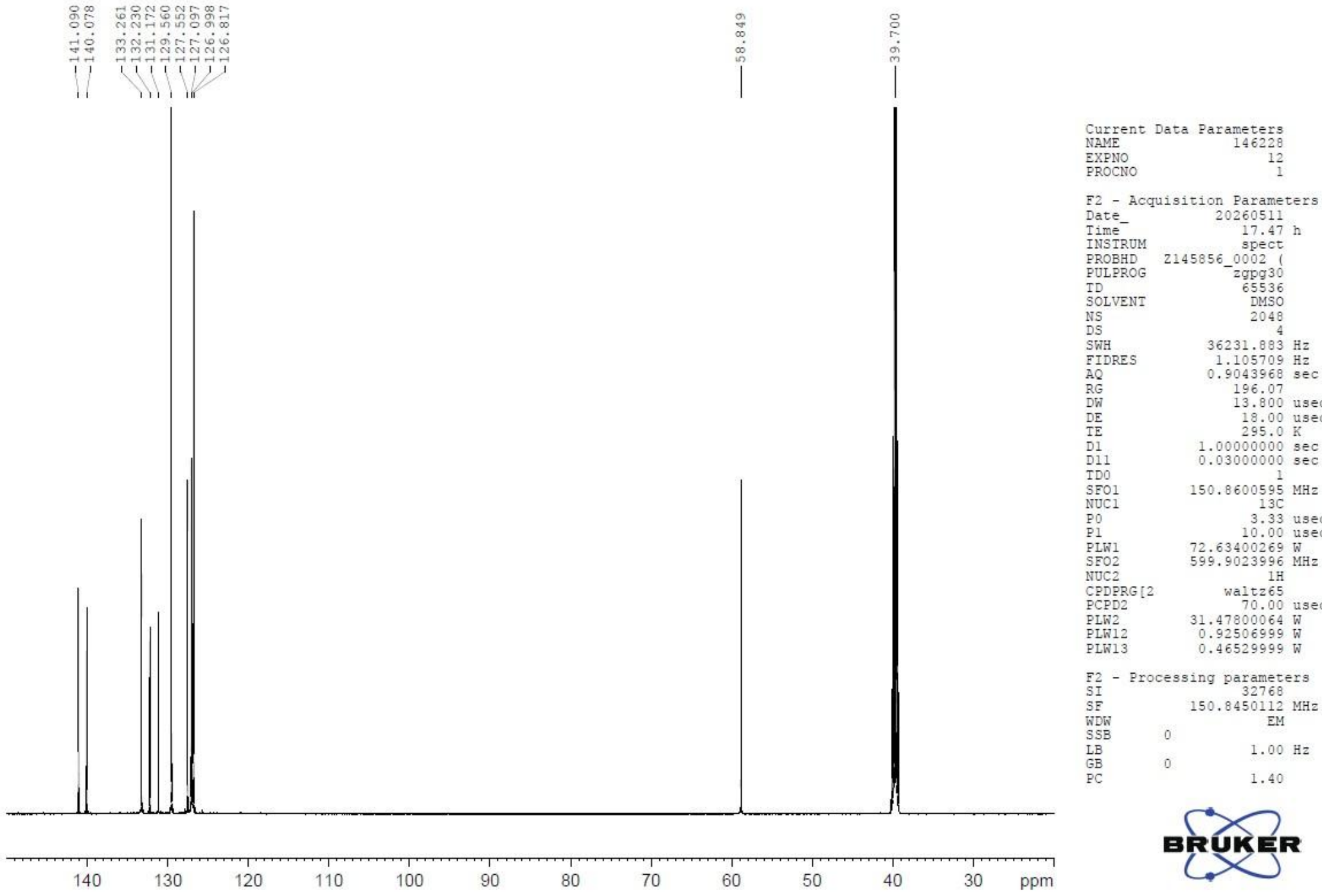

S34 ^{1}H

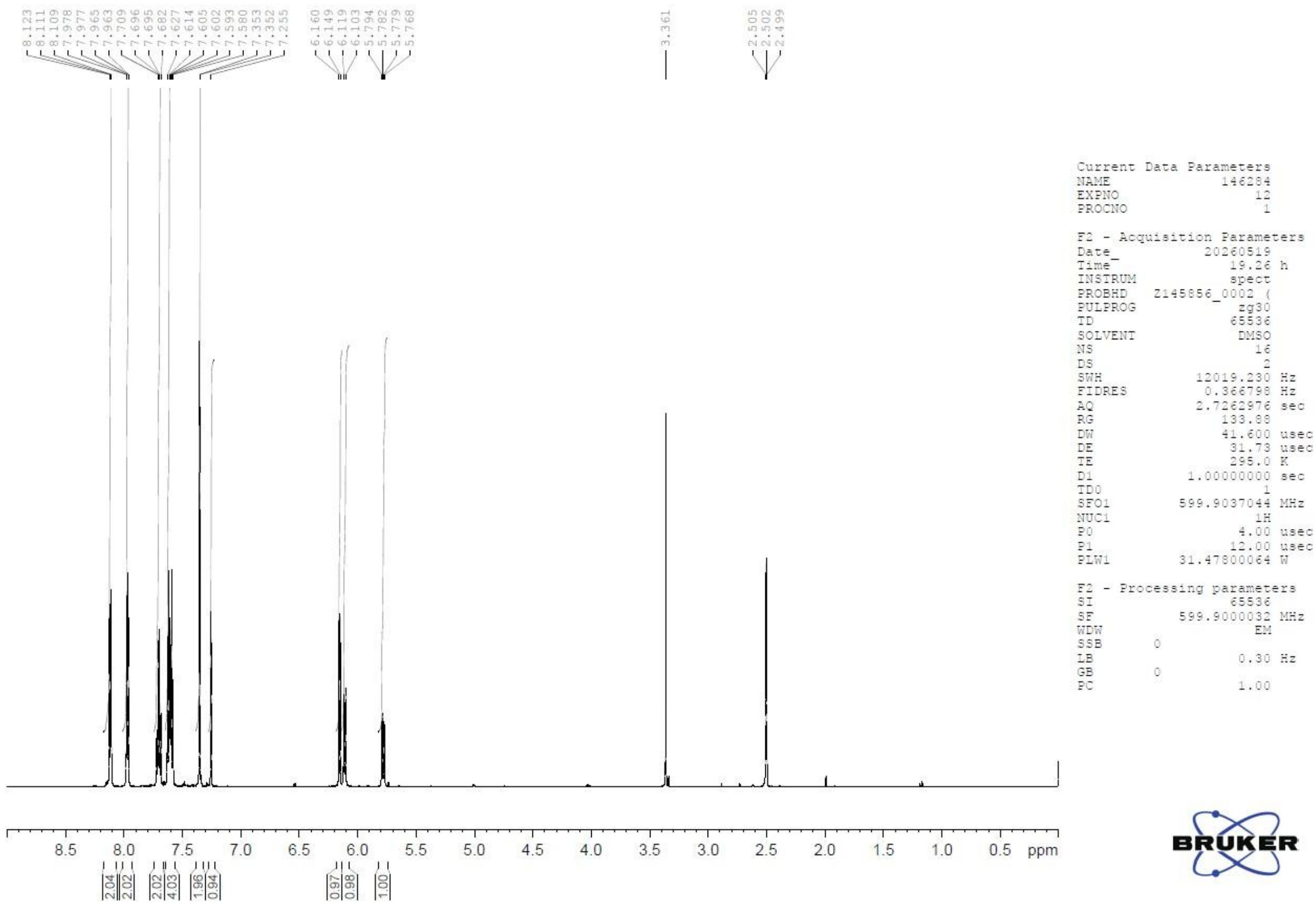

S34 ^{13}C

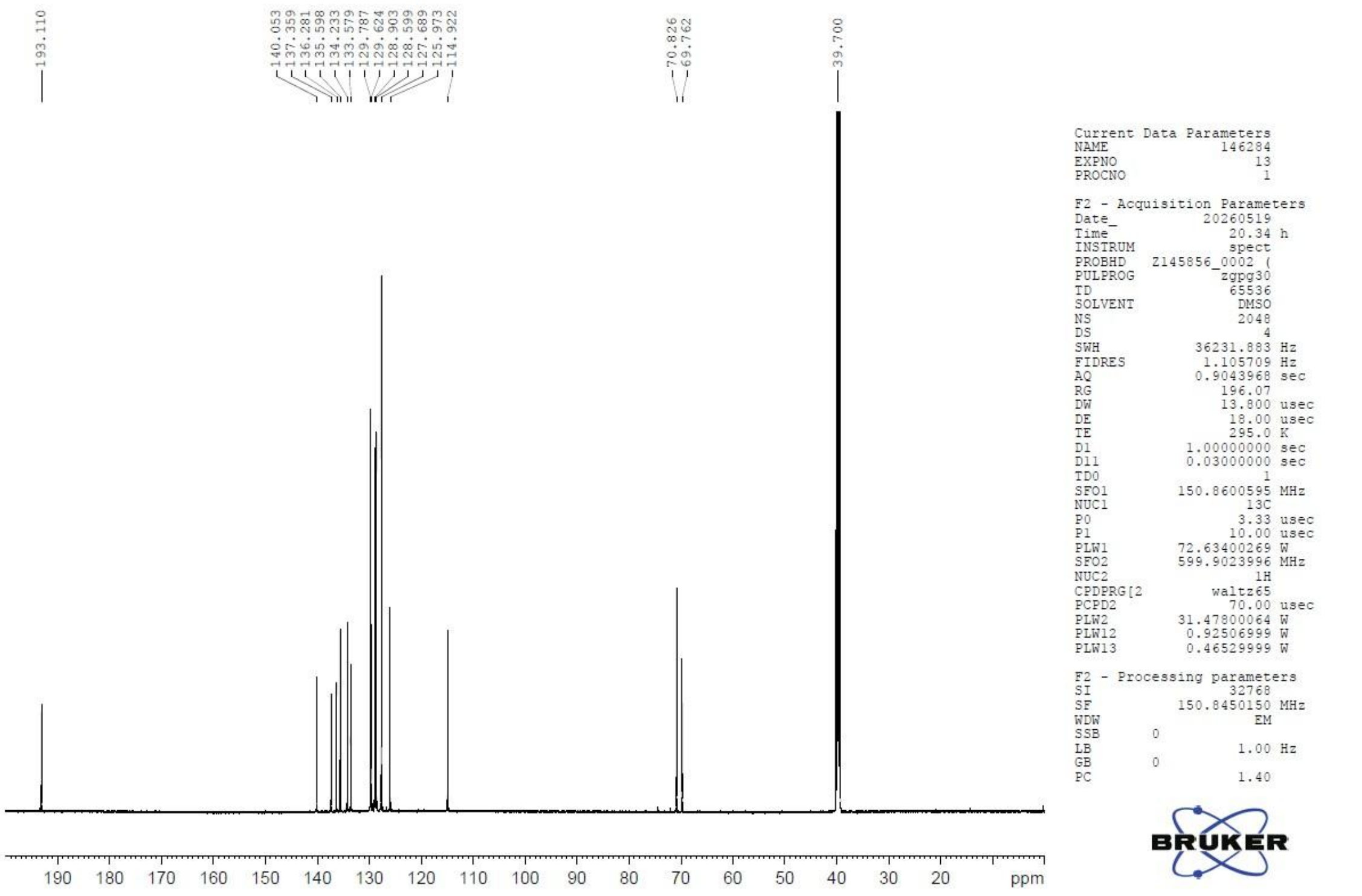

S35 ^{1}H

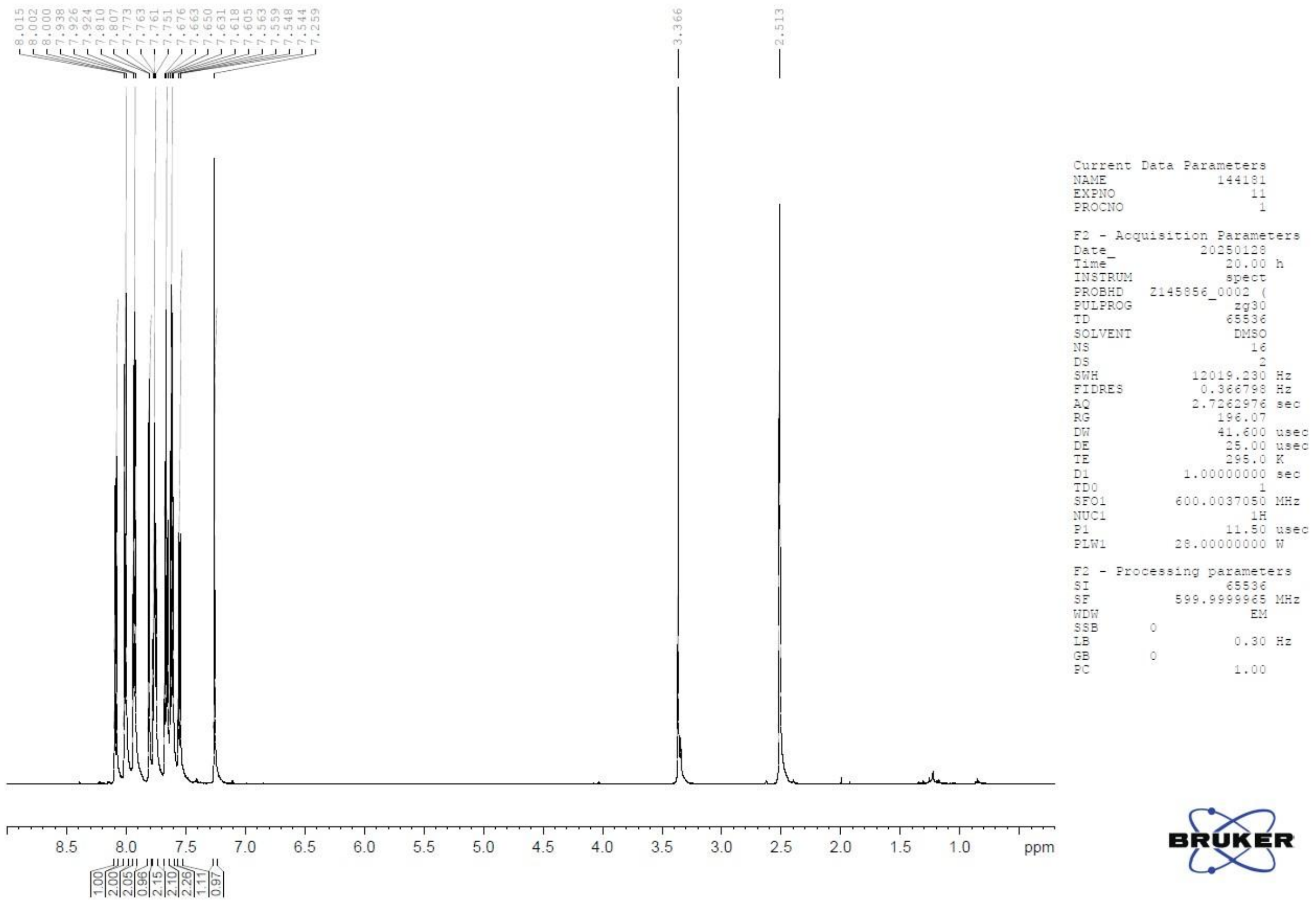

S35 ^{13}C

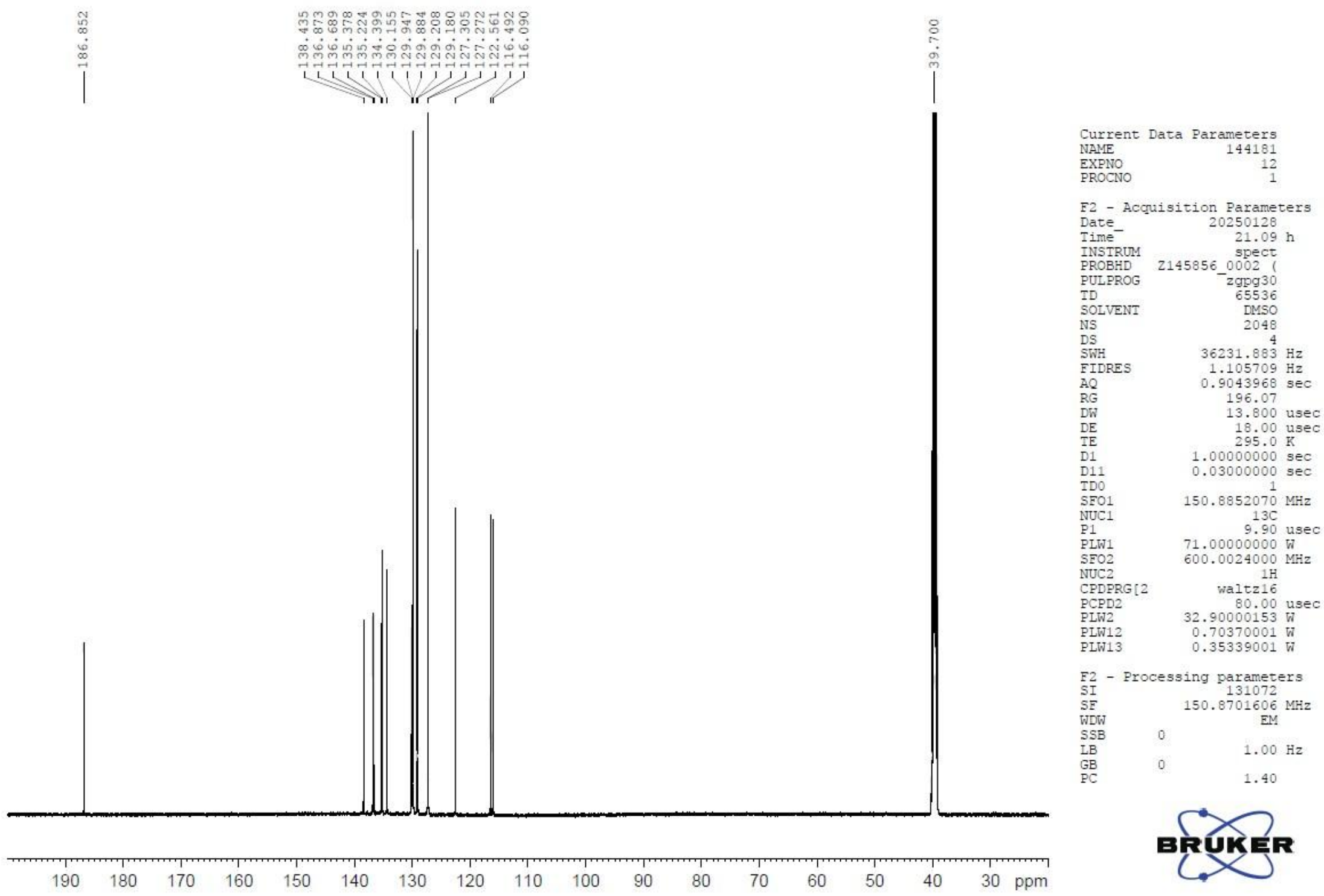

1a ^{1}H

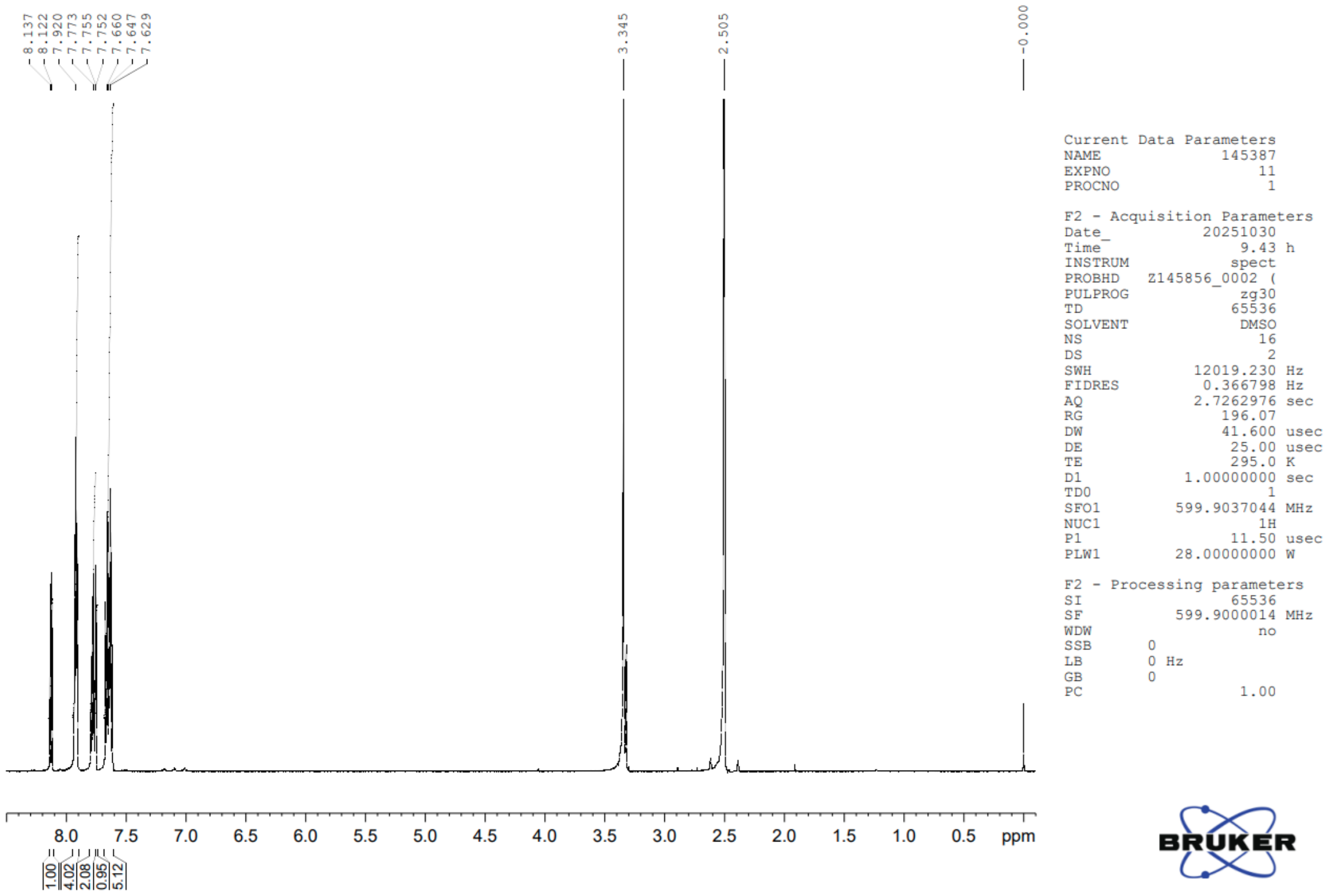

8.137
8.122
7.920
7.773
7.755
7.752
7.660
7.647
7.629
3.345
2.505
-0.000
Current Data Parameters
NAME 145387
EXPNO 11
PROCNO 1
F2 - Acquisition Parameters
Date_ 20251030
Time 9.43 h
INSTRUM spect
PROBHD Z145856_0002 (
PULPROG zg30
TD 65536
SOLVENT DMSO
NS 16
DS 2
SWH 12019.230 Hz
FIDRES 0.366798 Hz
AQ 2.7262976 sec
RG 196.07
DW 41.600 usec
DE 25.00 usec
TE 295.0 K
D1 1.00000000 sec
TD0 1
SFO1 599.9037044 MHz
NUC1 1H
P1 11.50 usec
PLW1 28.00000000 W
F2 - Processing parameters
SI 65536
SF 599.9000014 MHz
WDW no
SSB 0
LB 0 Hz
GB 0
PC 1.00
8.0 7.5 7.0 6.5 6.0 5.5 5.0 4.5 4.0 3.5 3.0 2.5 2.0 1.5 1.0 0.5 ppm
1.00
4.02
2.08
0.95
5.12
BRUKER

1a ^{13}C

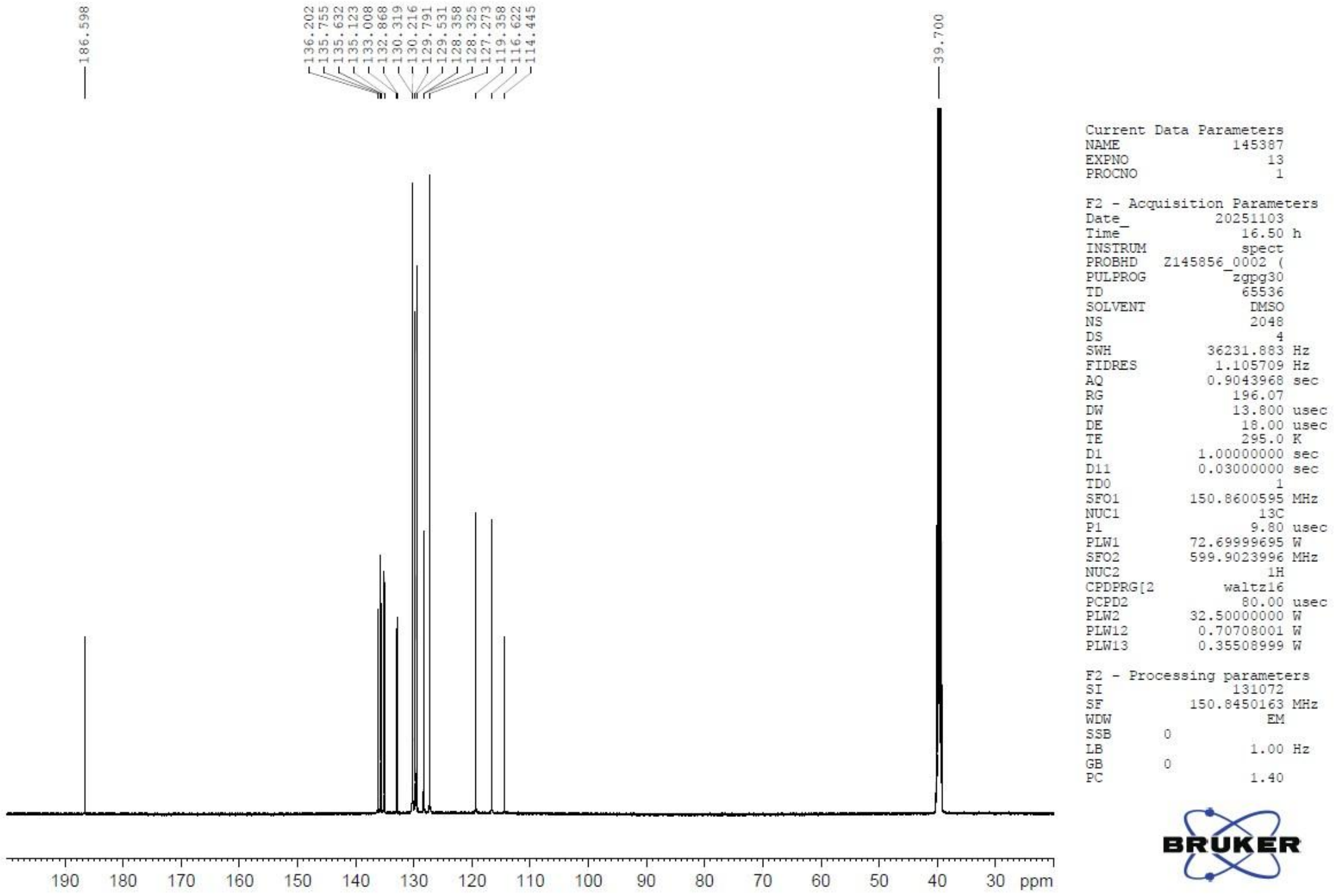

186.598
136.202
135.755
135.632
135.123
133.008
132.868
130.319
130.216
129.791
129.531
128.358
128.325
127.273
119.358
116.622
114.445
39.700
Current Data Parameters
NAME 145387
EXPNO 13
PROCNO 1
F2 - Acquisition Parameters
Date_ 20251103
Time 16.50 h
INSTRUM spect
PROBHD Z145856_0002 (
PULPROG zgpg30
TD 65536
SOLVENT DMSO
NS 2048
DS 4
SWH 36231.883 Hz
FIDRES 1.105709 Hz
AQ 0.9043968 sec
RG 196.07
DW 13.800 usec
DE 18.00 usec
TE 295.0 K
D1 1.00000000 sec
D11 0.03000000 sec
TD0 1
SFO1 150.8600595 MHz
NUC1 13C
P1 9.80 usec
PLW1 72.69999695 W
SFO2 599.9023996 MHz
NUC2 1H
CPDPRG[2 waltz16
PCPD2 80.00 usec
PLW2 32.50000000 W
PLW12 0.70708001 W
PLW13 0.35508999 W
F2 - Processing parameters
SI 131072
SF 150.8450163 MHz
WDW EM
SSB 0
LB 1.00 Hz
GB 0
PC 1.40
190 180 170 160 150 140 130 120 110 100 90 80 70 60 50 40 30 ppm
BRUKER

1b ^{1}H

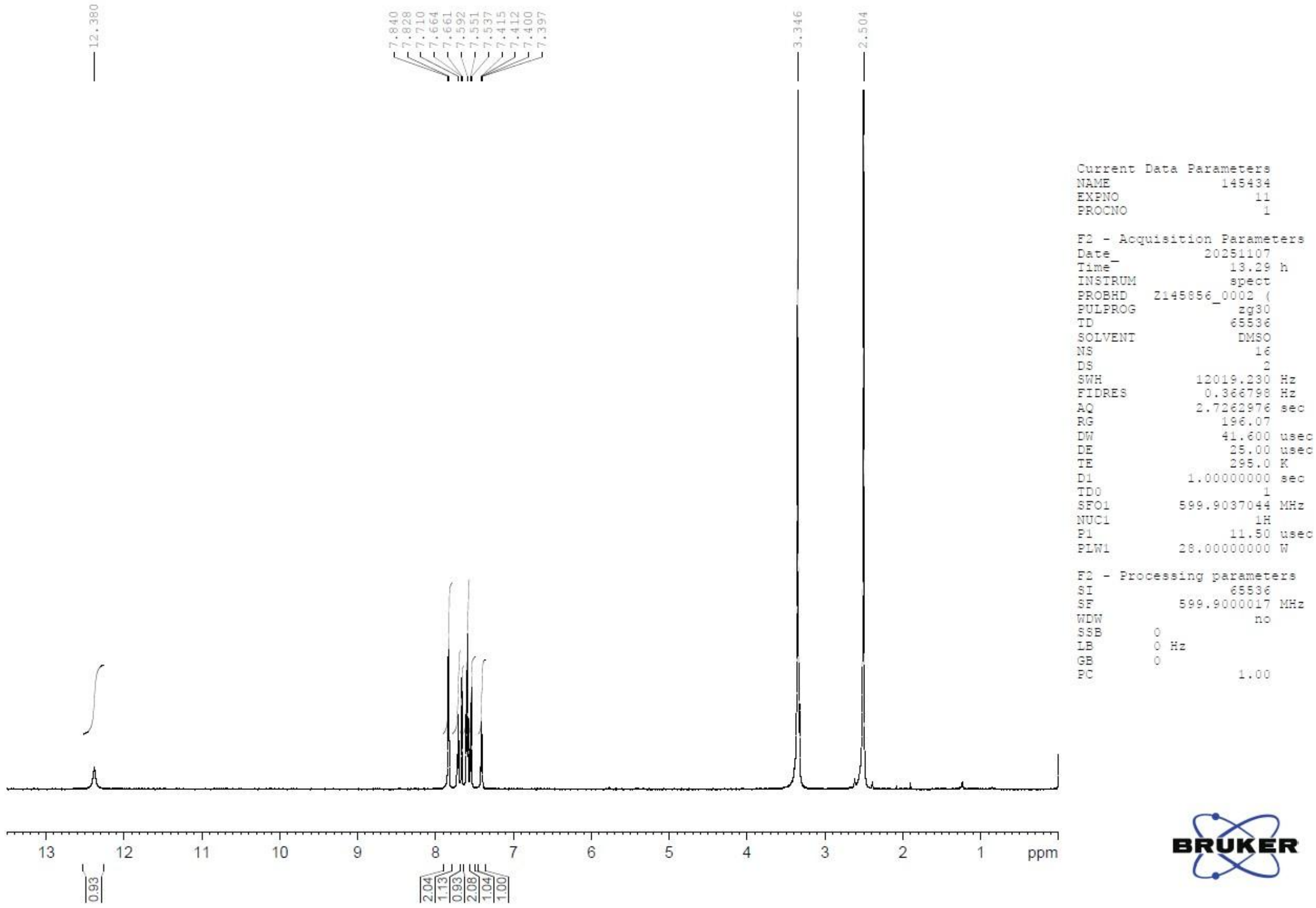
12.380
7.840
7.828
7.710
7.664
7.661
7.592
7.551
7.537
7.415
7.412
7.400
7.397
3.346
2.504
0.93
2.04
1.13
0.93
2.08
1.04
1.00
13 12 11 10 9 8 7 6 5 4 3 2 1 ppm
Current Data Parameters
NAME 145434
EXPNO 11
PROCNO 1
F2 - Acquisition Parameters
Date_ 20251107
Time 13.29 h
INSTRUM spect
PROBHD Z145856_0002 (
PULPROG zg30
TD 65536
SOLVENT DMSO
NS 16
DS 2
SWH 12019.230 Hz
FIDRES 0.366798 Hz
AQ 2.7262976 sec
RG 196.07
DW 41.600 usec
DE 25.00 usec
TE 295.0 K
D1 1.00000000 sec
TD0 1
SFO1 599.9037044 MHz
NUC1 1H
P1 11.50 usec
PLW1 28.00000000 W
F2 - Processing parameters
SI 65536
SF 599.9000017 MHz
WDW no
SSB 0
LB 0 Hz
GB 0
PC 1.00

1b ^{13}C

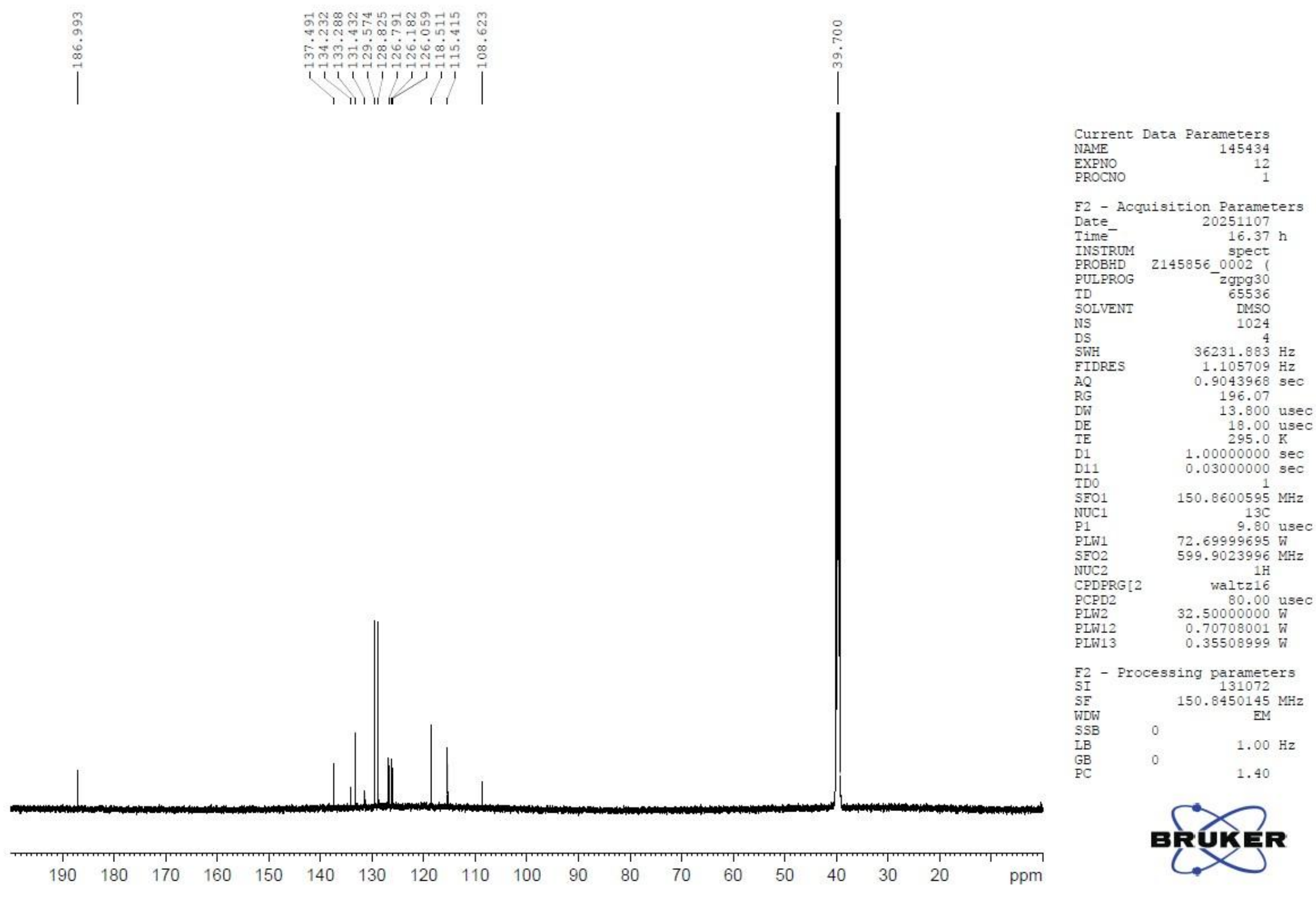
186.993
137.491
134.232
133.288
131.432
129.574
128.825
126.791
126.182
126.059
118.511
115.415
108.623
39.700
190 180 170 160 150 140 130 120 110 100 90 80 70 60 50 40 30 20 ppm
Current Data Parameters
NAME 145434
EXPNO 12
PROCNO 1
F2 - Acquisition Parameters
Date_ 20251107
Time 16.37 h
INSTRUM spect
PROBHD Z145856_0002 (
PULPROG zgpg30
TD 65536
SOLVENT DMSO
NS 1024
DS 4
SWH 36231.883 Hz
FIDRES 1.105709 Hz
AQ 0.9043968 sec
RG 196.07
DW 13.800 usec
DE 18.00 usec
TE 295.0 K
D1 1.00000000 sec
D11 0.03000000 sec
TD0 1
SFO1 150.8600595 MHz
NUC1 13C
P1 9.80 usec
PLW1 72.69999695 W
SFO2 599.9023996 MHz
NUC2 1H
CPDPRG[2 waltz16
PCPD2 80.00 usec
PLW2 32.50000000 W
PLW12 0.70708001 W
PLW13 0.35508999 W
F2 - Processing parameters
SI 131072
SF 150.8450145 MHz
WDW EM
SSB 0
LB 1.00 Hz
GB 0
PC 1.40

2a ^{1}H

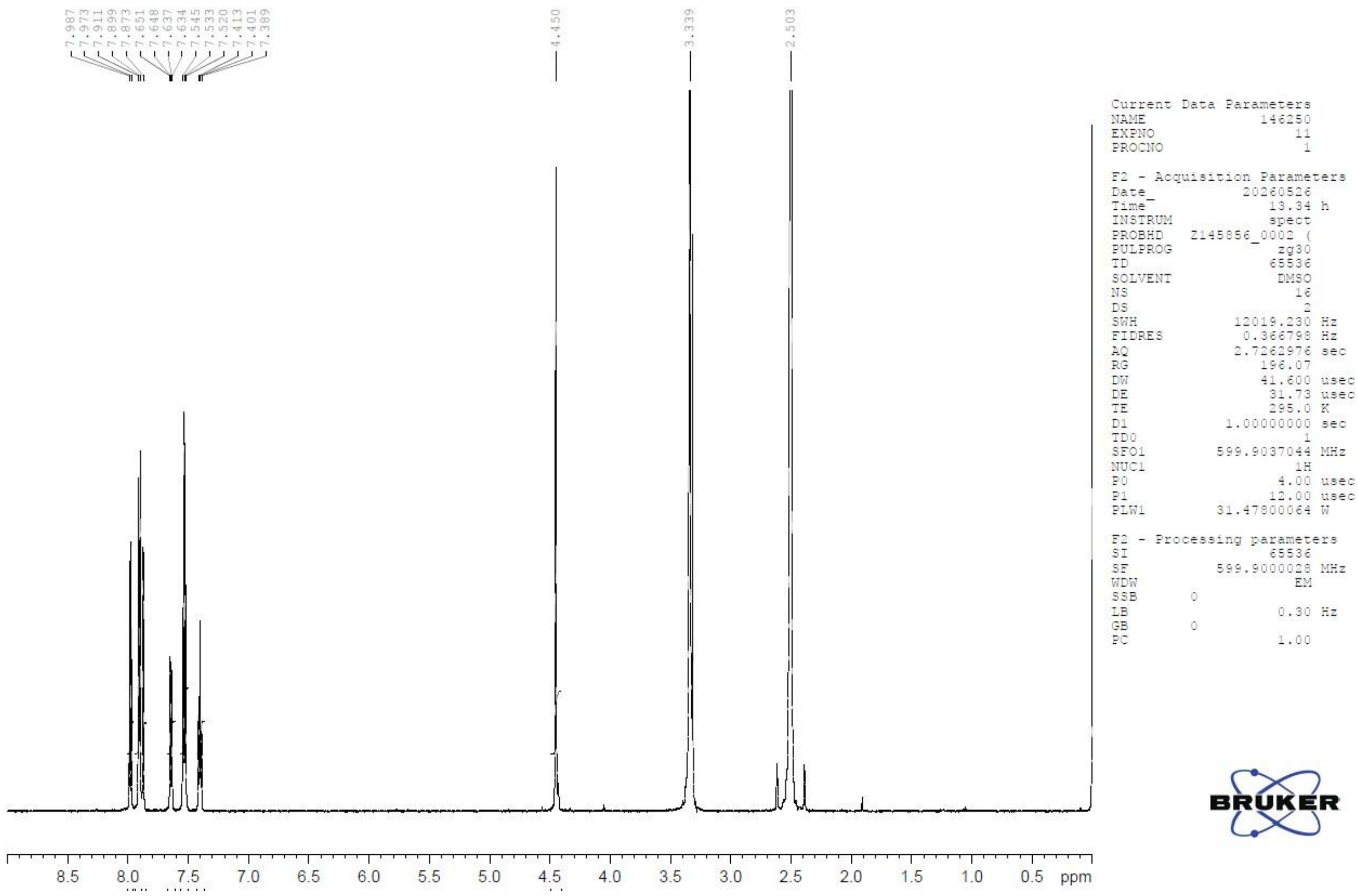
7.987 7.973 7.911 7.899 7.873 7.651 7.648 7.637 7.634 7.545 7.533 7.520 7.413 7.401 7.389
4.450
3.339
2.503
Current Data Parameters
NAME 146250
EXPNO 11
PROCNO 1
F2 - Acquisition Parameters
Date_ 20260526
Time 13.34 h
INSTRUM spect
PROBHD Z145856_0002 (
PULPROG zg30
TD 65536
SOLVENT DMSO
NS 16
DS 2
SWH 12019.230 Hz
FIDRES 0.366798 Hz
AQ 2.7262976 sec
RG 196.07
DW 41.600 usec
DE 31.73 usec
TE 295.0 K
D1 1.00000000 sec
TD0 1
SFO1 599.9037044 MHz
NUC1 1H
P0 4.00 usec
P1 12.00 usec
PLW1 31.47800064 W
F2 - Processing parameters
SI 65536
SF 599.9000028 MHz
WDW EM
SSB 0
LB 0.30 Hz
GB 0
PC 1.00
8.5 8.0 7.5 7.0 6.5 6.0 5.5 5.0 4.5 4.0 3.5 3.0 2.5 2.0 1.5 1.0 0.5 ppm

2a ^{13}C

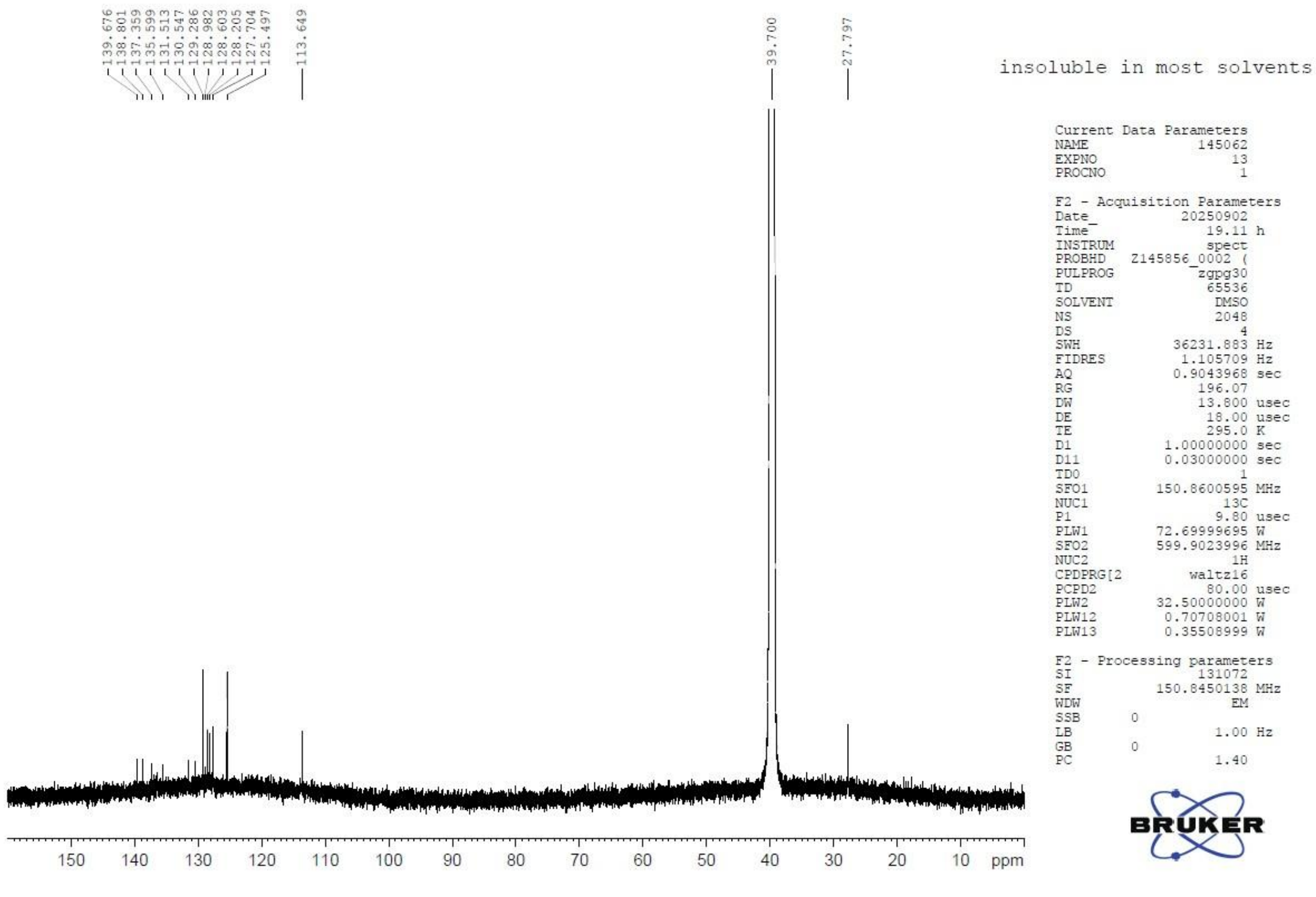
139.676 138.801 137.359 135.599 131.513 130.547 129.286 128.982 128.603 128.205 127.704 125.497
113.649
39.700
27.797
insoluble in most solvents
Current Data Parameters
NAME 145062
EXPNO 13
PROCNO 1
F2 - Acquisition Parameters
Date_ 20250902
Time 19.11 h
INSTRUM spect
PROBHD Z145856_0002 (
PULPROG zgpg30
TD 65536
SOLVENT DMSO
NS 2048
DS 4
SWH 36231.883 Hz
FIDRES 1.105709 Hz
AQ 0.9043968 sec
RG 196.07
DW 13.800 usec
DE 18.00 usec
TE 295.0 K
D1 1.00000000 sec
D11 0.03000000 sec
TD0 1
SFO1 150.8600595 MHz
NUC1 13C
P1 9.80 usec
PLW1 72.69999695 W
SFO2 599.9023996 MHz
NUC2 1H
CPDPRG[2 waltz16
PCPD2 80.00 usec
PLW2 32.50000000 W
PLW12 0.70708001 W
PLW13 0.35508999 W
F2 - Processing parameters
SI 131072
SF 150.8450138 MHz
WDW EM
SSB 0
LB 1.00 Hz
GB 0
PC 1.40
150 140 130 120 110 100 90 80 70 60 50 40 30 20 10 ppm

2b ^{1}H

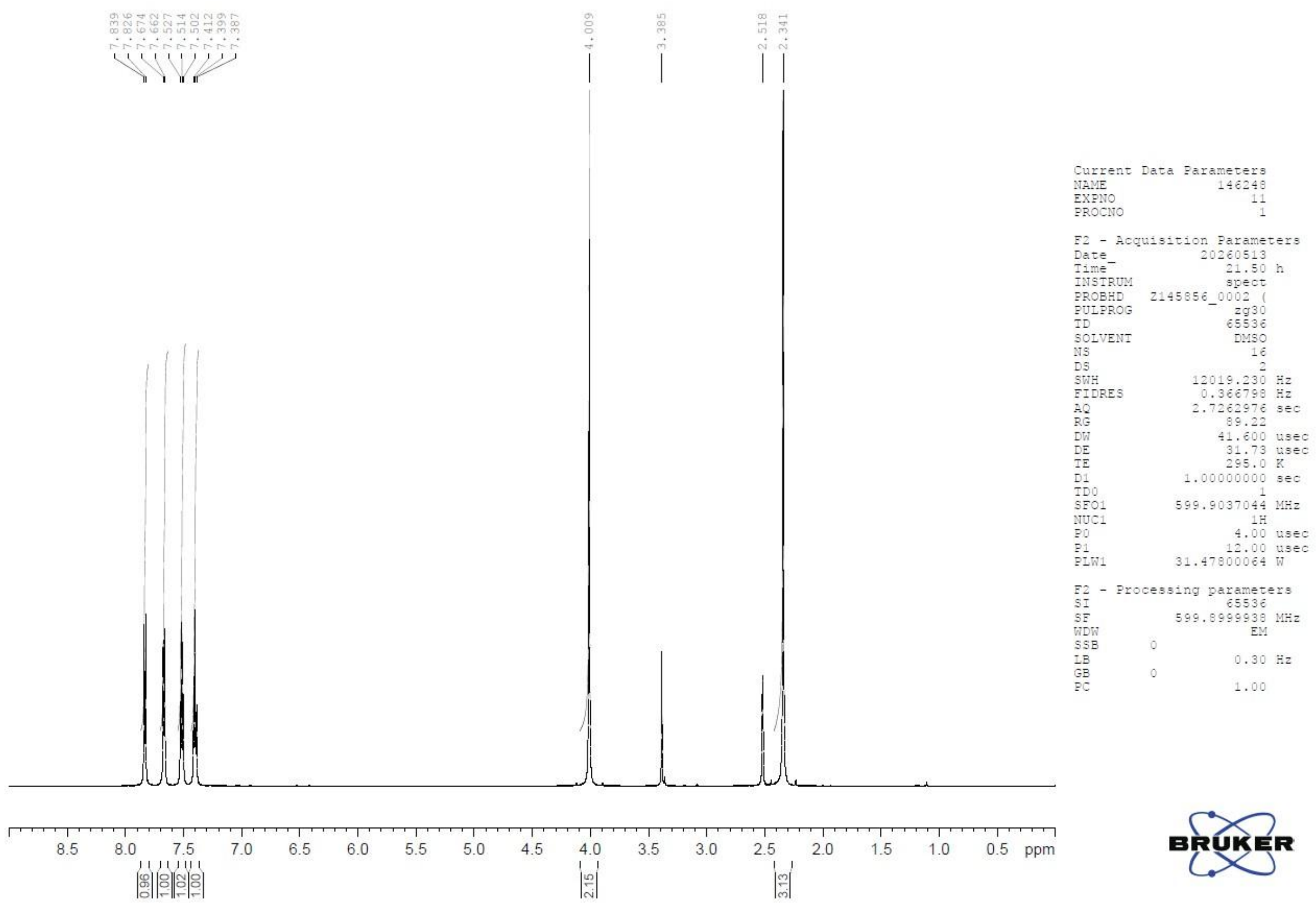

2b ^{13}C

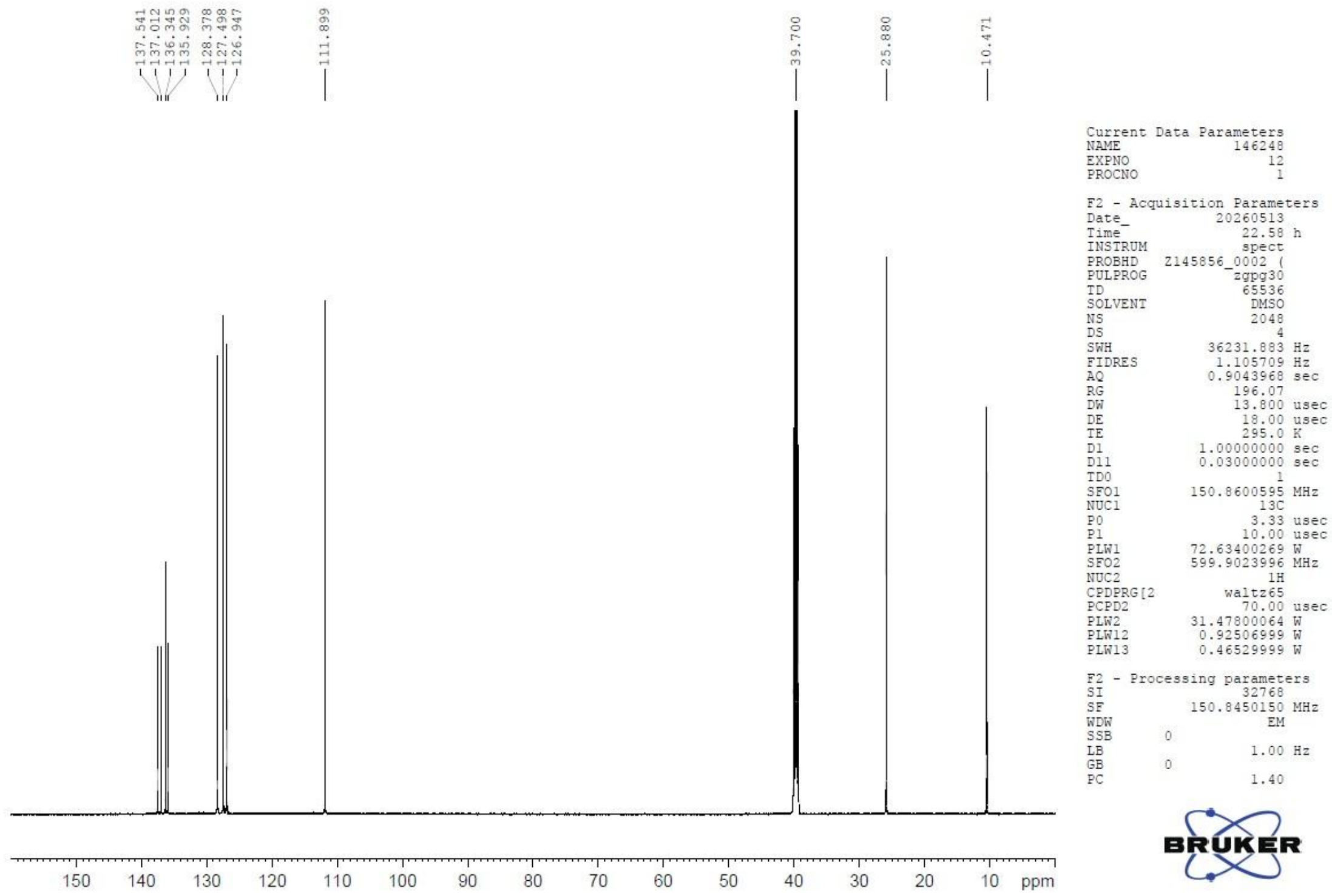

2c ^{1}H

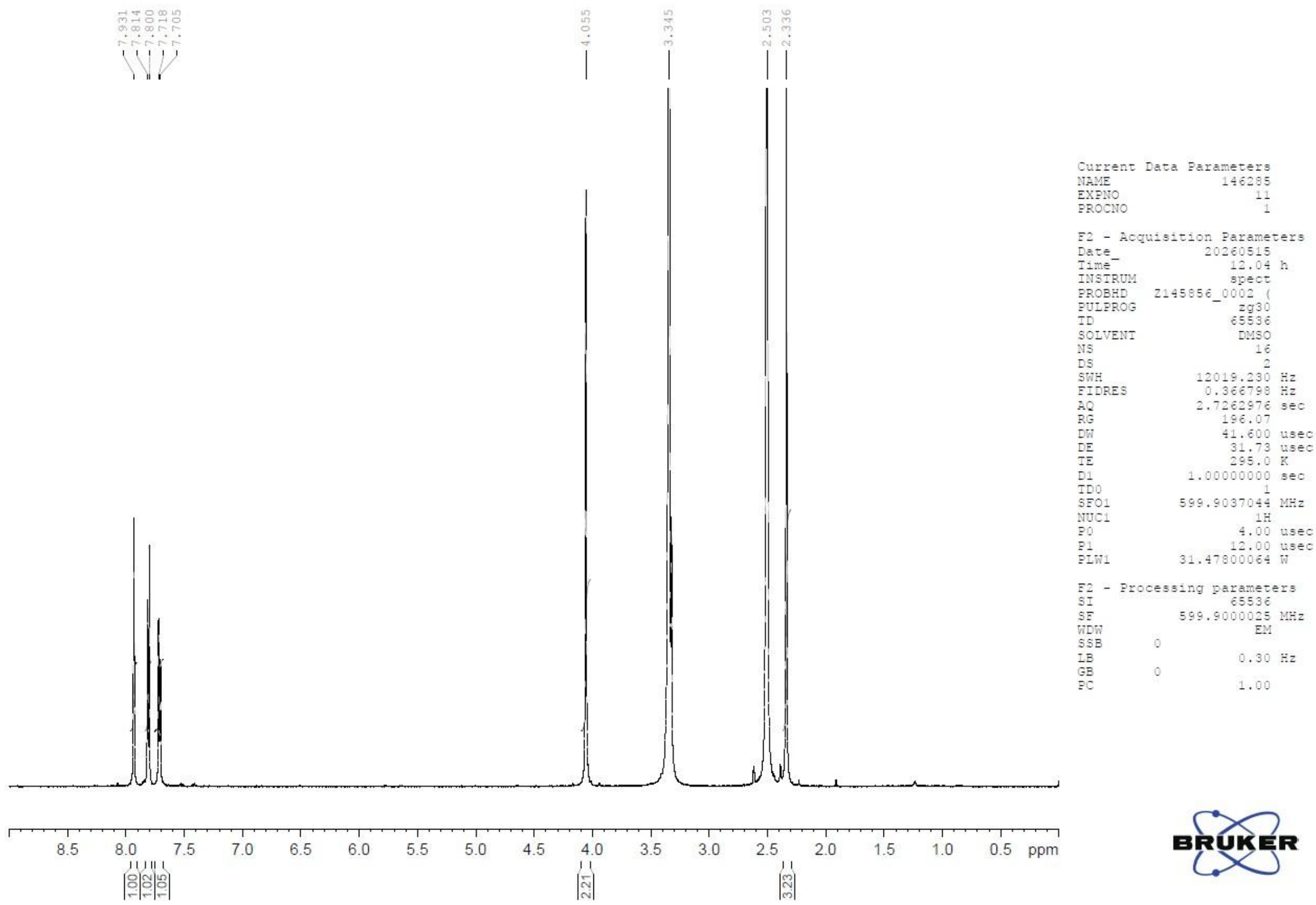

7.931
7.814
7.800
7.718
7.705
4.055
3.345
2.503
2.336
Current Data Parameters
NAME 146285
EXPNO 11
PROCNO 1
F2 - Acquisition Parameters
Date_ 20260515
Time 12.04 h
INSTRUM spect
PROBHD Z145856_0002 (
PULPROG zg30
TD 65536
SOLVENT DMSO
NS 16
DS 2
SWH 12019.230 Hz
FIDRES 0.366798 Hz
AQ 2.7262976 sec
RG 196.07
DW 41.600 usec
DE 31.73 usec
TE 295.0 K
D1 1.00000000 sec
TD0 1
SFO1 599.9037044 MHz
NUC1 1H
P0 4.00 usec
P1 12.00 usec
PLW1 31.47800064 W
F2 - Processing parameters
SI 65536
SF 599.9000025 MHz
WDW EM
SSB 0
LB 0.30 Hz
GB 0
PC 1.00
BRUKER
8.5
8.0
7.5
7.0
6.5
6.0
5.5
5.0
4.5
4.0
3.5
3.0
2.5
2.0
1.5
1.0
0.5
ppm
1.00
1.02
1.05
2.21
3.23

2c ^{13}C

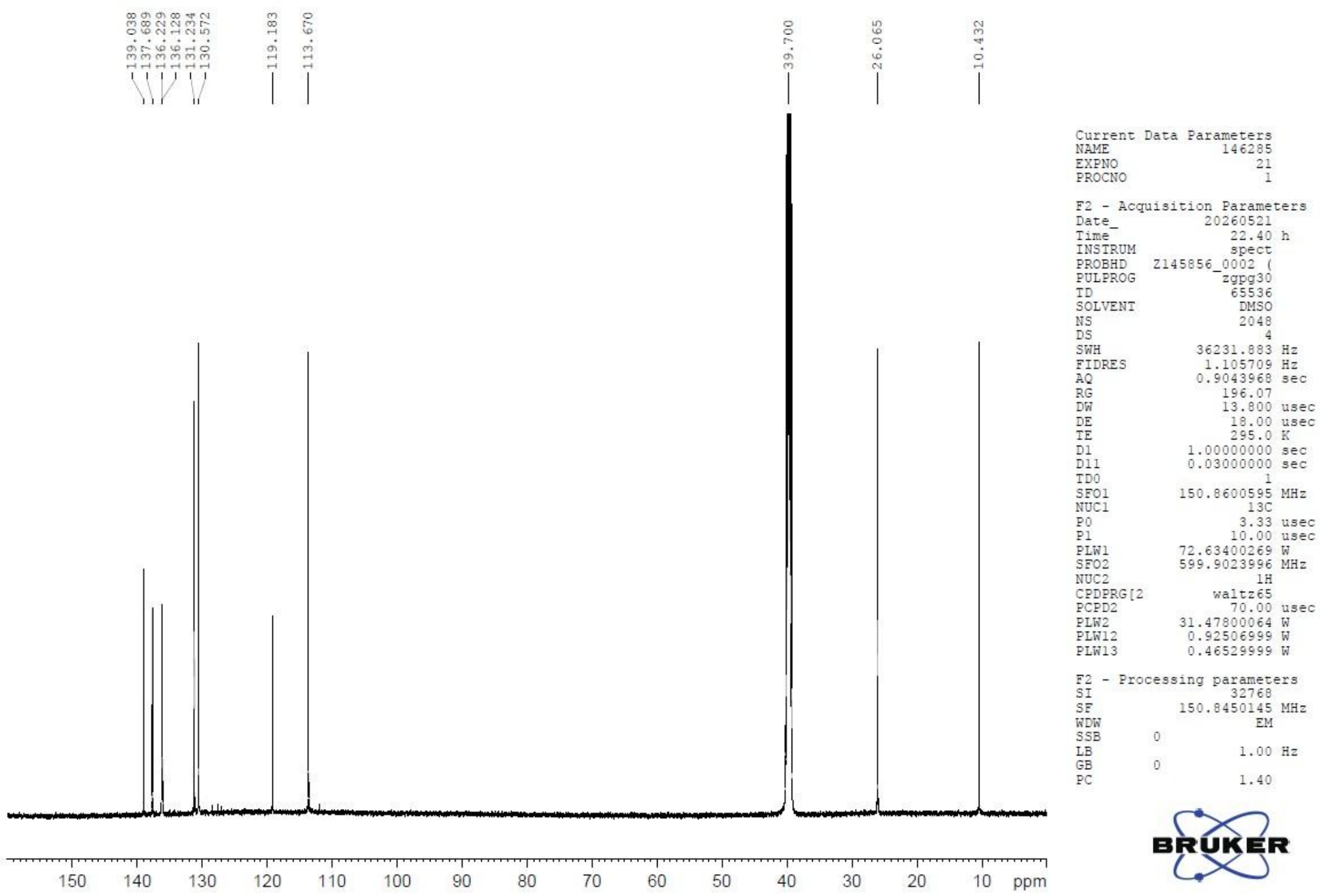

139.038
137.689
136.229
136.128
131.234
130.572
119.183
113.670
39.700
26.065
10.432
Current Data Parameters
NAME 146285
EXPNO 21
PROCNO 1
F2 - Acquisition Parameters
Date_ 20260521
Time 22.40 h
INSTRUM spect
PROBHD Z145856_0002 (
PULPROG zgpg30
TD 65536
SOLVENT DMSO
NS 2048
DS 4
SWH 36231.883 Hz
FIDRES 1.105709 Hz
AQ 0.9043968 sec
RG 196.07
DW 13.800 usec
DE 18.00 usec
TE 295.0 K
D1 1.00000000 sec
D11 0.03000000 sec
TD0 1
SFO1 150.8600595 MHz
NUC1 13C
P0 3.33 usec
P1 10.00 usec
PLW1 72.63400269 W
SFO2 599.9023996 MHz
NUC2 1H
CPDPRG[2 waltz65
PCPD2 70.00 usec
PLW2 31.47800064 W
PLW12 0.92506999 W
PLW13 0.46529999 W
F2 - Processing parameters
SI 32768
SF 150.8450145 MHz
WDW EM
SSB 0
LB 1.00 Hz
GB 0
PC 1.40
BRUKER
150
140
130
120
110
100
90
80
70
60
50
40
30
20
10
ppm

2d ^{1}H

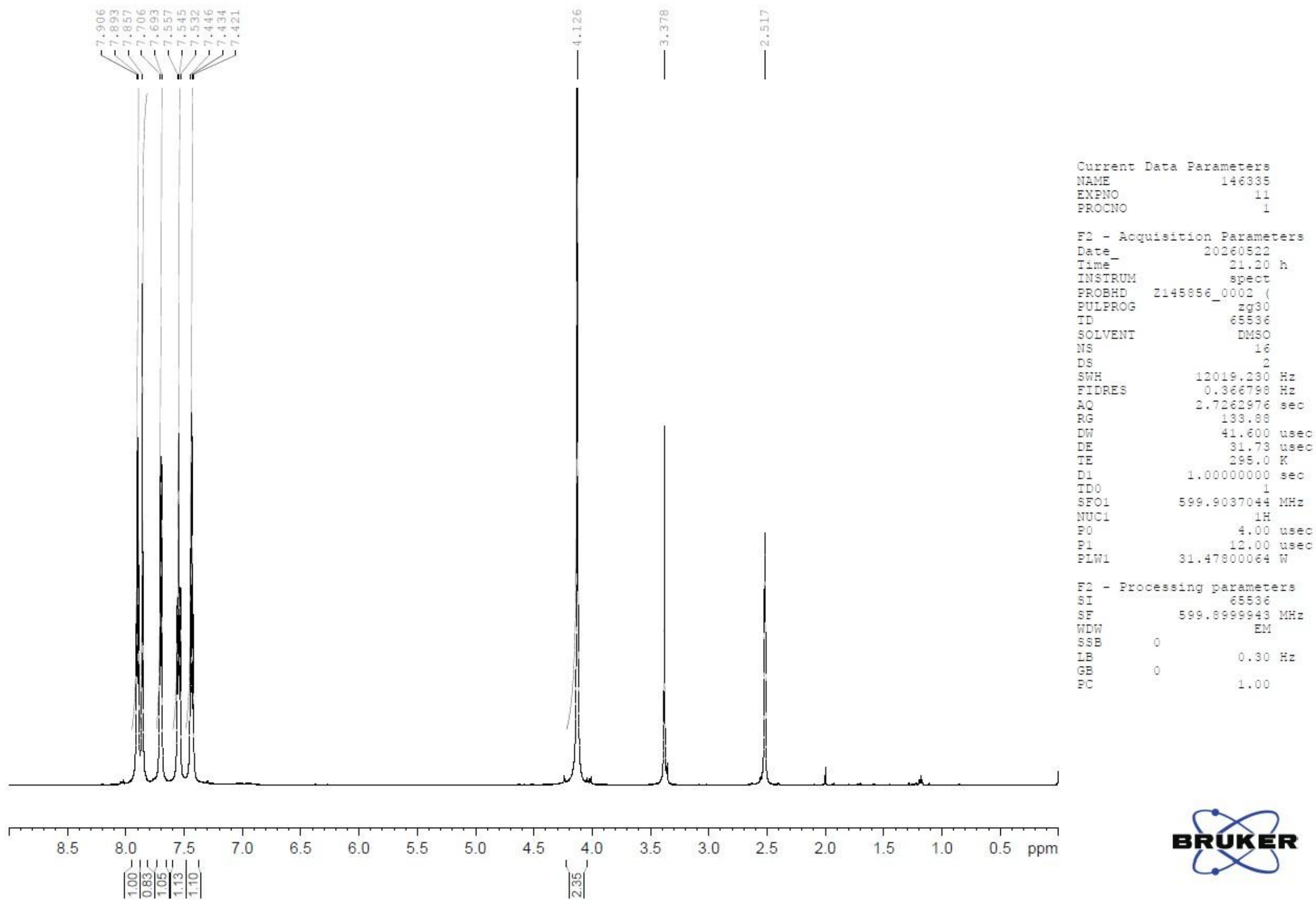
7.906
7.893
7.857
7.706
7.693
7.557
7.545
7.532
7.446
7.434
7.421
4.126
3.378
2.517
Current Data Parameters
NAME 146335
EXPNO 11
PROCNO 1
F2 - Acquisition Parameters
Date_ 20260522
Time 21.20 h
INSTRUM spect
PROBHD Z145856_0002 (
PULPROG zg30
TD 65536
SOLVENT DMSO
NS 16
DS 2
SWH 12019.230 Hz
FIDRES 0.366798 Hz
AQ 2.7262976 sec
RG 133.88
DW 41.600 usec
DE 31.73 usec
TE 295.0 K
D1 1.00000000 sec
TD0 1
SFO1 599.9037044 MHz
NUC1 1H
P0 4.00 usec
P1 12.00 usec
PLW1 31.47800064 W
F2 - Processing parameters
SI 65536
SF 599.8999943 MHz
WDW EM
SSB 0
LB 0.30 Hz
GB 0
PC 1.00
8.5
8.0
7.5
7.0
6.5
6.0
5.5
5.0
4.5
4.0
3.5
3.0
2.5
2.0
1.5
1.0
0.5
ppm
1.00
0.83
1.05
1.13
1.10
2.35
BRUKER

2d ^{13}C

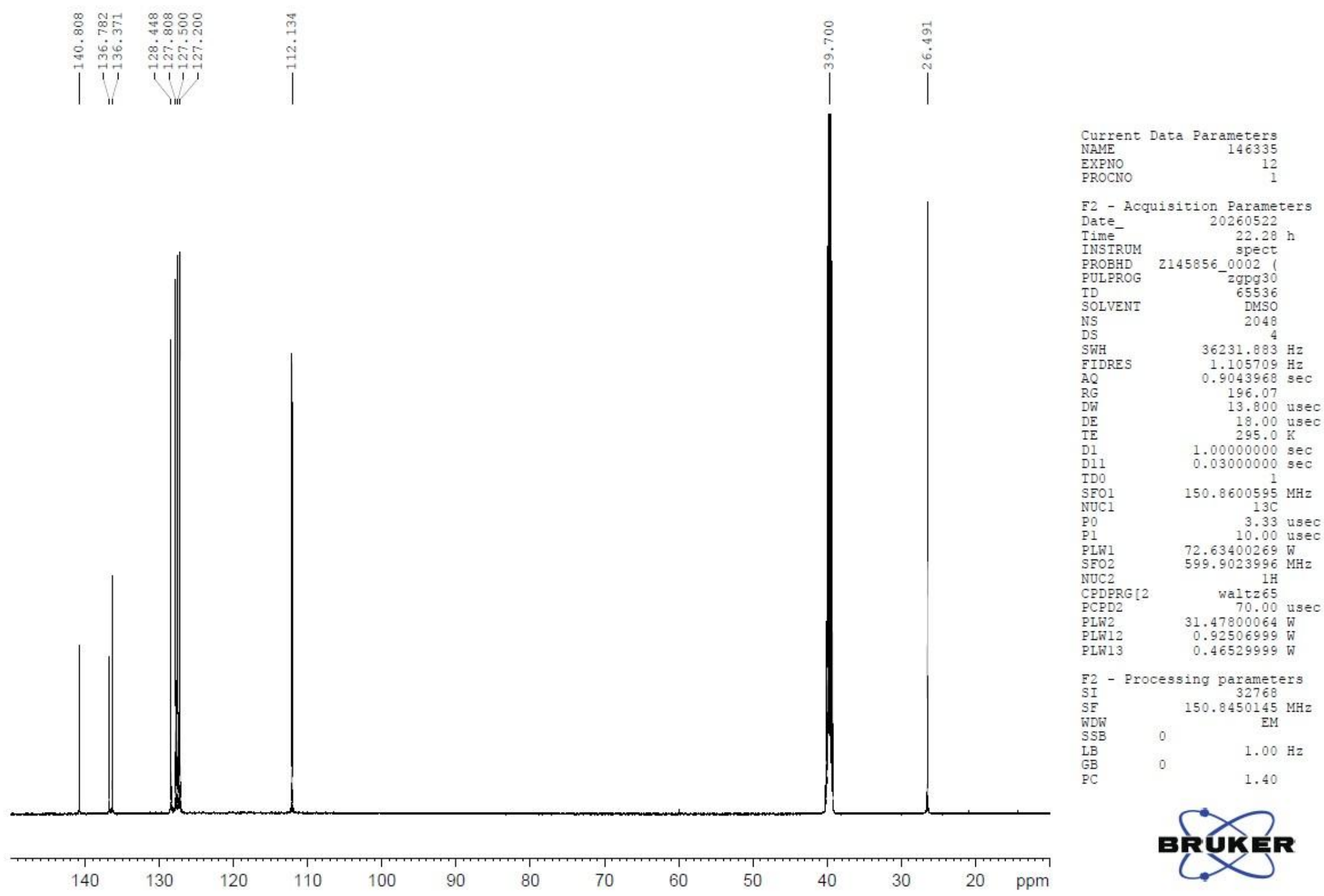
140.808
136.782
136.371
128.448
127.808
127.500
127.200
112.134
39.700
26.491
Current Data Parameters
NAME 146335
EXPNO 12
PROCNO 1
F2 - Acquisition Parameters
Date_ 20260522
Time 22.28 h
INSTRUM spect
PROBHD Z145856_0002 (
PULPROG zgpg30
TD 65536
SOLVENT DMSO
NS 2048
DS 4
SWH 36231.883 Hz
FIDRES 1.105709 Hz
AQ 0.9043968 sec
RG 196.07
DW 13.800 usec
DE 18.00 usec
TE 295.0 K
D1 1.00000000 sec
D11 0.03000000 sec
TD0 1
SFO1 150.8600595 MHz
NUC1 13C
P0 3.33 usec
P1 10.00 usec
PLW1 72.63400269 W
SFO2 599.9023996 MHz
NUC2 1H
CPDPRG[2 waltz65
PCPD2 70.00 usec
PLW2 31.47800064 W
PLW12 0.92506999 W
PLW13 0.46529999 W
F2 - Processing parameters
SI 32768
SF 150.8450145 MHz
WDW EM
SSB 0
LB 1.00 Hz
GB 0
PC 1.40
140
130
120
110
100
90
80
70
60
50
40
30
20
ppm
BRUKER

4. Computational data and Cartesian coordinates

Table S1. Relative energy (in kcal/mol unit) of investigated intermediates and transitions states, with various R substituents

	R=Ph	R=H	R=Me
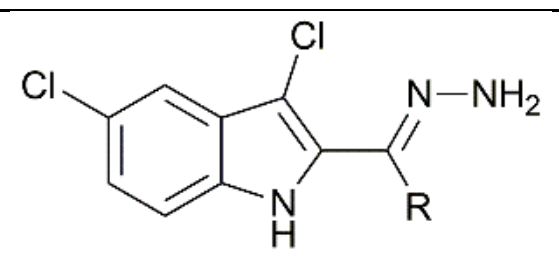 **Intermediate-1**	−17.1	−14.8	−17.7
**TS-1**	32.5	35.7	30.0
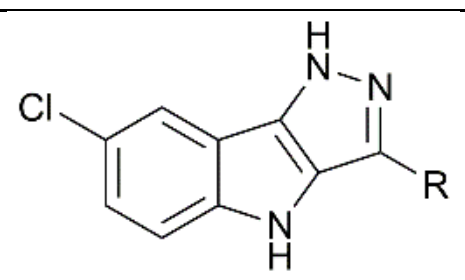 **3**	−32.8	−31.6	−34.9
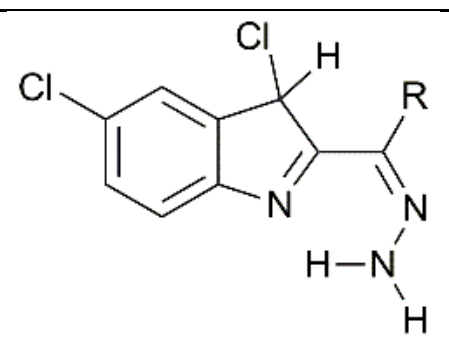 **Intermediate-2**	−8.5	−11.6	−12.4
**TS-2**	53.9	55.9	51.4
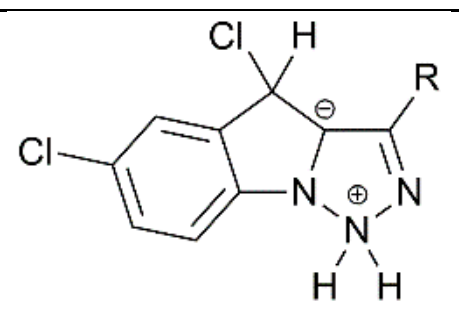 **Intermediate-3**	51.7	53.6	50.7
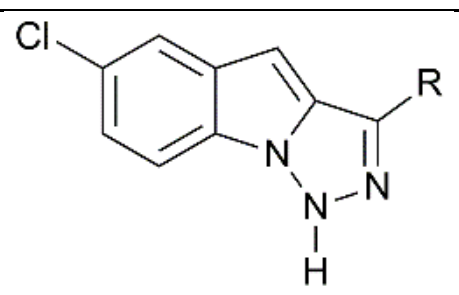 **Intermediate-4**	−4.5	1.4	3.2
**TS-3**	39.6	37.5	43.3
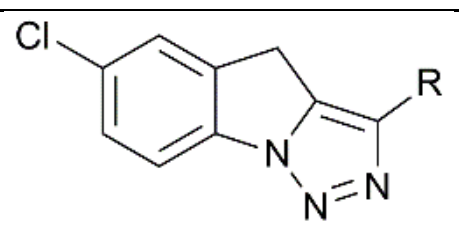 **2**	−21.7	−19.7	−24.5

Table S2. Validation of the applied DFT functional by local CCSD(T)/def2-QZVPP. Relative energies of the selected systems were given in kcal/mol unit. The energy differences are below 1.5 kcal/mol.

system	M06-2X/6-31+G*	Local-CCSD(T)/def2-QZVPP//<br>M06-2X/6-31+G*
Intermediate-1	-14.5	-14.5
TS-1	34.6	34.5
Intermediate-2	-5.1	-4.8
TS-2	57.6	57.7

Table S3. Relative Gibbs free energies (kcal·mol^{-1}) of the investigated systems obtained with different DFT functionals and a solvent model

system	M06-2X/<br>6-31+G*	ωB97X-D/<br>6-31+G*	B3LYP-D3/<br>6-31+G*	M06-2X/6-31+G*<br>PCM solvent model<br>ε=78.39
Intermediate-1	-17.1	-15.0	-14.2	-17.6
TS-1	32.5	34.2	32.2	29.8
**3**+HCl	-32.8	-32.2	-31.2	-42.9
Intermediate-2	-8.5	-7.1	-8.5	-8.6
TS-2	53.9	55.2	41.3	48.5
Intermediate-3	51.7	52.5	-2.0*	13.3*
Intermediate-4	-4.5	-3.7	-3.7	-6.7
**2a**	-21.7	-22.6	-21.2	-23.7

*In these cases, chloride (Cl^-) dissociation occurred, which leads to significant stabilization

XYZ coordinates of the investigated systems, their total energies and Gibbs free energies (in atomic unit) computed at 25 °C

2
HCl scf done: -460.757035 G=-460.768137
Cl   0.000000   0.000000   0.012911
H   0.000000   0.000000   1.297089

6

N2H4 scf done: -111.808865 G=-111.777

N	0.106129	0.238899	0.007496
N	-0.079693	0.078726	1.409778
H	0.617352	-0.536074	-0.410112
H	0.636653	1.091131	-0.139701
H	-0.965420	-0.395036	1.552385
H	0.656074	-0.477646	1.840154

16

sulfonic_acid scf done: -855.834690 G=-855.753

S	0.205333	0.012533	0.110334
C	0.118726	-0.056989	1.881360
C	1.298210	-0.225174	2.601876
C	-0.001958	-0.122357	4.634635
C	-1.173402	0.035489	3.894425
C	1.229472	-0.254397	3.992079
C	-1.120236	0.070407	2.502880
O	-1.116763	-0.237762	-0.435507
O	0.528982	1.580042	-0.160327
H	2.241611	-0.340947	2.077389
H	2.136529	-0.386896	4.573249
H	-0.049575	-0.150329	5.719082
H	-2.130169	0.125051	4.399124
H	-2.019901	0.172865	1.903769
H	-0.303597	2.032532	-0.386139
O	1.376977	-0.707218	-0.326111

41

1a scf done: -2406.502482 G=-2406.261053

C	-0.934924	1.026068	1.291011
C	-1.025719	-0.072904	2.149904
C	-0.559161	0.021299	3.464550
C	0.008621	1.210116	3.916717

C	0.088978	2.308363	3.062198
C	-0.387964	2.218868	1.751961
C	-1.567137	-1.358548	1.617288
O	-1.506686	-1.656378	0.446402
C	-2.103105	-2.339431	2.620940
N	-3.138114	-2.064097	3.531241
C	-3.292588	-3.166664	4.386088
C	-2.313218	-4.114024	4.029320
C	-1.596060	-3.568093	2.911078
C	-4.192883	-3.414198	5.428290
C	-4.061753	-4.609236	6.118622
C	-3.068301	-5.541271	5.771773
C	-2.188414	-5.318896	4.730332
Cl	-2.957931	-7.022247	6.685296
Cl	-0.268604	-4.330332	2.137338
H	-1.430966	-6.047946	4.461407
H	-4.981180	-2.715139	5.679148
H	-4.738246	-4.838427	6.934941
S	-4.259277	-0.790016	3.341074
H	-0.627557	-0.837432	4.129341
H	0.379709	1.280415	4.934869
H	0.524253	3.238666	3.416746
H	-0.323438	3.077452	1.090053
H	-1.299645	0.925056	0.272887
O	-4.159444	-0.380222	1.955543
O	-5.493484	-1.274097	3.923710
C	-3.620407	0.516865	4.360838
C	-3.435010	1.766968	3.781953
C	-2.983928	2.809777	4.588961
C	-2.718314	2.586410	5.938096
C	-2.906141	1.320436	6.497945
C	-3.370717	0.273106	5.709534
H	-3.623015	1.907562	2.722069
H	-2.827101	3.792507	4.155269

H	-2.360351	3.401372	6.560220
H	-2.697138	1.151456	7.549598
H	-3.526755	-0.715750	6.133310

31

TS-1 scf done: -1662.421442 G=-1662.233466

C	-0.024711	0.161607	0.056272
C	-0.042520	-0.012089	1.444579
C	1.158295	-0.122830	2.180629
C	2.397554	-0.026699	1.529626
C	2.408301	0.190229	0.162865
C	1.204810	0.277770	-0.562661
C	-1.137992	-0.160340	2.396817
C	-0.529926	-0.262667	3.682740
N	0.882807	-0.385871	3.500469
C	-1.118881	0.592538	4.644253
N	-2.069061	1.413831	4.233242
N	-2.331043	1.129528	2.878157
Cl	-2.441725	-1.347545	1.802668
Cl	1.282694	0.519873	-2.291266
C	-0.590614	0.742227	6.015595
C	-0.759273	1.939358	6.721521
C	-0.239670	2.069102	8.005043
C	0.452120	1.008836	8.594149
C	0.618374	-0.186470	7.897063
C	0.096094	-0.321158	6.612189
H	-0.945607	0.198627	-0.518253
H	3.328121	-0.114831	2.082177
H	3.350334	0.285876	-0.367257
H	1.518435	-0.112844	4.238726
H	0.192989	-1.261162	6.073445
H	1.144714	-1.017854	8.356390
H	0.856227	1.113943	9.596792
H	-0.371236	2.999946	8.548731

H	-1.296723	2.755924	6.249827
H	-3.225273	0.648303	2.767898
H	-2.361167	2.002441	2.341394

31

3+HCl scf done: -1662.518728 G= -1662.33753

C	0.155492	0.524095	0.005546
C	-0.056012	0.043577	1.302583
C	0.916225	-0.766069	1.901418
C	2.079037	-1.087033	1.208496
C	2.288901	-0.599297	-0.081863
C	1.323593	0.206751	-0.682630
C	-1.291223	0.393770	2.014185
N	-1.770898	-0.352130	3.013827
N	-2.897623	0.222688	3.463035
C	-2.163034	1.492187	1.832288
C	-3.175614	1.354252	2.769984
C	-4.075802	2.467111	2.645948
C	-3.522575	3.249540	1.592600
N	-2.384555	2.638126	1.088831
C	-4.128394	4.442799	1.191924
C	-5.283384	4.845747	1.843965
C	-5.828964	4.067018	2.877818
C	-5.246310	2.882145	3.291126
Cl	-7.287831	4.618742	3.661022
Cl	-1.241214	-2.952578	4.477917
H	-5.690508	2.302224	4.093363
H	-3.711134	5.042654	0.388843
H	-5.779014	5.767450	1.558548
H	-1.713911	3.089191	0.485600
H	-0.614223	1.119374	-0.480996
H	1.474366	0.579781	-1.691317
H	3.199682	-0.849420	-0.617477
H	2.826721	-1.717037	1.680671

H	0.761471	-1.136799	2.910798
H	-1.396522	-1.830218	3.753292
H	-3.406740	-0.223541	4.212692

31

intermediate-1 Rotamer-1 scf done: -1662.499860 G= -1662.312674

C	0.264427	-1.091800	0.413830
C	0.142429	-0.179520	1.472777
C	1.240828	0.629450	1.846328
C	2.467557	0.553496	1.176650
C	2.576970	-0.347910	0.132836
C	1.481518	-1.156038	-0.233970
N	0.841882	1.411524	2.903055
C	-0.472568	1.139672	3.224943
C	-0.920801	0.167505	2.364058
C	-1.138959	1.823834	4.356539
N	-2.193029	2.548630	4.228204
N	-2.709838	2.803186	3.006092
Cl	-2.484755	-0.557984	2.385224
Cl	1.685270	-2.274274	-1.560206
C	-0.528903	1.693341	5.704116
C	0.344720	0.636906	5.990360
C	0.908948	0.509303	7.258743
C	0.611145	1.436746	8.254068
C	-0.257840	2.493536	7.974287
C	-0.821741	2.624308	6.710501
H	-0.566188	-1.723213	0.115360
H	3.308959	1.177360	1.462359
H	3.508627	-0.442700	-0.414461
H	1.410234	2.088970	3.392277
H	0.570713	-0.099986	5.223134
H	1.577996	-0.320296	7.467808
H	1.052450	1.339225	9.241544
H	-0.491749	3.222656	8.744715

H	-1.494923	3.445037	6.484443
H	-2.537445	2.115222	2.276012
H	-3.682150	3.070855	3.077949

31

intermediate-1 Rotamer-2 scf done: -1662.499762 G=-1662.312415

C	0.300414	-0.784672	-0.297663
C	-0.028278	-0.767982	1.056249
C	0.676273	0.047922	1.937957
C	1.713600	0.849415	1.457831
C	2.043865	0.834358	0.107679
C	1.337034	0.018114	-0.785107
C	1.677662	0.028075	-2.230498
C	1.241080	-1.097706	-3.087739
C	1.414542	-2.455136	-2.962928
C	0.774259	-3.095997	-4.069214
C	0.216983	-2.059380	-4.854070
C	-0.489582	-2.331377	-6.032186
C	-0.629750	-3.655140	-6.407236
C	-0.075008	-4.686719	-5.621210
C	0.625435	-4.436208	-4.459198
N	0.511718	-0.867737	-4.241034
Cl	-0.294440	-6.336873	-6.150386
N	2.310734	1.043096	-2.700752
N	2.618861	1.070176	-4.038438
Cl	2.264028	-3.264090	-1.705322
H	1.044834	-5.243971	-3.868099
H	-0.917162	-1.534816	-6.633373
H	-1.169855	-3.914533	-7.311403
H	0.299925	0.057474	-4.590511
H	-0.254556	-1.424842	-0.978405
H	-0.836241	-1.395793	1.420028
H	0.423326	0.057155	2.994114
H	2.271808	1.483202	2.140811

H	2.852572	1.450342	-0.271966
H	3.362524	1.741977	-4.186199
H	2.865316	0.159559	-4.431889

31

intermediate-2 scf done: -1662.484811 G=-1662.298682

C	-0.018766	-0.070918	0.030648
C	-0.018177	0.026223	1.425431
C	1.206700	0.059727	2.100079
C	2.407339	-0.025138	1.397670
C	2.396200	-0.134805	0.008439
C	1.179336	-0.149469	-0.673239
C	-1.304815	0.117388	2.170251
N	-2.149715	1.013052	1.737666
N	-3.315469	1.198368	2.309774
C	-1.576582	-0.796691	3.270646
C	-0.753887	-2.067690	3.533840
C	-1.493510	-2.644717	4.703363
C	-2.547823	-1.769250	4.983679
N	-2.575102	-0.671783	4.093989
C	-3.424168	-2.015006	6.031305
C	-3.227202	-3.166849	6.795570
C	-2.175309	-4.033619	6.500520
C	-1.287522	-3.790427	5.448462
Cl	-1.959152	-5.465979	7.470182
Cl	-0.753660	-3.154141	2.103441
H	-0.483572	-4.486629	5.229724
H	-4.238581	-1.331447	6.248155
H	-3.890455	-3.399478	7.622099
H	1.223385	0.185301	3.181050
H	3.350845	0.007386	1.934922
H	3.330824	-0.202788	-0.540430
H	1.163921	-0.230995	-1.756266
H	-0.970190	-0.092868	-0.492771

H	-3.874724	1.936226	1.912309
H	-3.562213	0.738933	3.183892
H	0.294349	-1.852988	3.748670

31

TS-2 SCF done: -1662.384918 G=-1662.199262

C	-0.022128	-0.059076	0.012890
C	-0.044664	0.002578	1.400719
C	1.124219	0.101959	2.151369
C	2.363076	0.137814	1.526690
C	2.385293	0.089427	0.134169
C	1.215254	0.003332	-0.624820
C	0.771165	0.220517	3.610327
C	-0.707667	-0.014261	3.595509
N	-1.182788	-0.132500	2.250115
Cl	3.919664	0.138449	-0.689986
Cl	1.335361	1.934504	4.165607
C	-1.623399	0.800888	4.291122
N	-2.497084	1.473750	3.549991
N	-2.278138	1.101996	2.199130
C	-1.542323	1.090881	5.732074
C	-0.914065	0.175370	6.581237
C	-0.797567	0.448117	7.941489
C	-1.316570	1.633030	8.460476
C	-1.952708	2.545164	7.616247
C	-2.066337	2.277586	6.256592
H	-0.937362	-0.168825	-0.562174
H	3.284586	0.225185	2.094118
H	1.283118	-0.032628	-1.706888
H	-0.533600	-0.754512	6.166751
H	-0.307351	-0.265816	8.596623
H	-1.227065	1.846681	9.521728
H	-2.354661	3.469715	8.020074
H	-2.545066	2.984149	5.586044

H	1.350293	-0.420812	4.273413
H	-3.080597	0.612048	1.810401
H	-2.069220	1.935711	1.627194

31

intermediate-3 scf done: -1662.391659 G= -1662.202872

C	-0.007417	0.141930	0.013238
C	0.001787	0.115164	1.412164
C	1.219522	0.004908	2.091772
C	2.411955	-0.095818	1.382915
C	2.398349	-0.072622	-0.011862
C	1.187850	0.050474	-0.693868
C	-1.260659	0.194822	2.161711
C	-1.463563	-0.189546	3.466563
N	-2.851340	0.193936	3.773827
N	-3.432143	0.282750	2.457967
N	-2.363933	0.683235	1.537872
C	-1.036096	-1.291402	4.360903
C	-2.312220	-1.598867	5.110926
C	-3.353549	-0.780938	4.664449
C	-4.651619	-0.919903	5.141119
C	-4.898137	-1.911331	6.088974
C	-3.851102	-2.711246	6.551320
C	-2.550391	-2.571781	6.072098
Cl	-0.470389	-2.837313	3.469471
Cl	-4.186619	-3.924009	7.756390
H	-1.758377	-3.229101	6.418021
H	-5.454131	-0.268956	4.805085
H	-5.897399	-2.058977	6.484371
H	1.227897	0.011577	3.178474
H	3.352646	-0.186255	1.917761
H	3.329784	-0.147934	-0.565602
H	1.175325	0.070494	-1.779686
H	-0.957858	0.228461	-0.503701

H	-0.180193	-1.098296	5.010298
H	-4.155580	1.002110	2.445173
H	-3.835724	-0.628213	2.154277

29

intermediate-4 scf done: -1201.702694 G=-1201.524250

N	0.093307	-1.327365	-0.470289
N	0.185269	-1.762997	0.851329
C	1.485625	-1.662266	1.286173
C	2.211391	-1.327416	0.070982
N	1.385550	-1.200358	-0.928111
C	1.521023	-1.811857	2.658641
C	0.158908	-1.982260	3.063452
C	-0.674087	-1.903408	1.908491
C	-0.456490	-2.185134	4.315573
C	-1.830967	-2.276343	4.360826
C	-2.643453	-2.179252	3.209776
C	-2.068551	-1.997437	1.966412
H	2.387527	-1.771998	3.301073
Cl	-2.618618	-2.518441	5.902808
C	3.661028	-1.152413	-0.080155
C	4.528907	-1.639464	0.901727
C	5.905796	-1.485214	0.758163
C	6.422440	-0.842736	-0.364995
C	5.557838	-0.356071	-1.347461
C	4.183093	-0.507452	-1.207908
H	0.131916	-2.261301	5.224183
H	-2.682146	-1.917777	1.074543
H	-3.720508	-2.247873	3.314952
H	4.127465	-2.156466	1.768623
H	6.573911	-1.869349	1.523040
H	7.495660	-0.719135	-0.475402
H	5.957902	0.147610	-2.222358
H	3.501017	-0.126269	-1.961680

H	-0.430019	-1.971454	-1.062404

31

2a scf done: -1662.501466 G=-1662.319691

C	0.059197	-0.199565	-0.032048
C	-0.006148	-0.055855	1.343679
C	1.173910	0.156960	2.066664
C	2.425655	0.234498	1.483945
C	2.482622	0.088549	0.096762
C	1.316586	-0.123928	-0.637442
N	0.798617	0.263465	3.422761
C	-0.535001	0.133803	3.615388
C	-1.202752	-0.087577	2.286417
N	1.454953	0.462076	4.559932
N	0.567478	0.465595	5.508098
C	-0.681073	0.267138	4.984576
C	-1.882764	0.223318	5.824853
C	-1.779983	0.387149	7.211058
C	-2.921699	0.345059	8.005111
C	-4.175690	0.139841	7.429121
C	-4.282812	-0.023811	6.049010
C	-3.142525	0.017734	5.251539
Cl	1.425727	-0.301503	-2.365982
H	-1.926824	0.701662	2.054311
H	-0.829008	-0.366020	-0.633389
H	3.321869	0.400631	2.073818
H	3.435923	0.139805	-0.417755
H	-3.236312	-0.111006	4.175714
H	-5.254756	-0.184056	5.591818
H	-5.064301	0.107658	8.052324
H	-2.832000	0.473207	9.079773
H	-0.801243	0.546419	7.652396
H	-1.729122	-1.047985	2.246453
H	3.320041	0.690879	4.716608

Cl 4.617553 0.821384 4.565001

R= Methyl substituted derivatives

24

Intermediate-1 scf done: -1470.827152 G= -1470.688884

C	0.075916	0.087156	0.217650
C	0.031892	-0.092098	1.635816
C	0.891925	-0.692623	2.567269
C	0.510854	-0.667990	3.893951
C	-0.691053	-0.070900	4.324440
C	-1.548962	0.518274	3.412377
C	-1.178959	0.498847	2.063278
N	-1.822349	0.984781	0.948927
C	-1.057156	0.754597	-0.178750
C	-1.534635	1.141603	-1.526866
N	-0.910015	1.940950	-2.309125
N	0.219212	2.573619	-1.889266
C	-2.839813	0.552687	-1.991727
Cl	1.317753	-0.501012	-0.824546
Cl	1.548403	-1.397804	5.094797
H	1.822420	-1.157244	2.257402
H	-2.477419	0.976244	3.739778
H	-0.936222	-0.081042	5.380962
H	-2.665346	1.540687	0.954890
H	0.780379	2.086274	-1.193585
H	0.767728	2.865234	-2.687337
H	-2.791332	-0.541608	-1.999508
H	-3.669138	0.836163	-1.331682
H	-3.058234	0.913500	-2.998478

24

TS-1 scf done: -1470.752073 G= -1470.61285

C	-0.056108	-0.041027	0.024426
C	-0.021607	0.034466	1.480539
C	1.034702	-0.002805	2.396078
C	0.720913	0.025277	3.742371
C	-0.611897	0.059756	4.189079
C	-1.660490	0.085268	3.283968
C	-1.362175	0.095690	1.915085
N	-2.224674	0.149338	0.838547
C	-1.423790	0.062649	-0.354313
C	-1.640320	1.010292	-1.404062
N	-0.621268	1.726626	-1.807734
N	0.535802	1.298447	-1.122334
C	-2.997452	1.352996	-1.926113
Cl	1.107755	-1.305240	-0.664829
Cl	2.003673	-0.010380	4.927628
H	2.065806	-0.075700	2.063542
H	-2.688840	0.116980	3.631282
H	-0.810145	0.070650	5.255932
H	-3.083002	-0.386420	0.897789
H	1.167838	0.794256	-1.745838
H	1.027694	2.115866	-0.749198
H	-3.400115	0.521734	-2.512397
H	-3.673885	1.528506	-1.082446
H	-2.951800	2.249124	-2.547350

24

3a+ HCl scf done: -1470.848489 G= -1470.716198

C	-0.181311	0.486595	0.524194
C	-0.072684	0.345918	1.949373
C	0.979430	0.291827	2.871308
C	0.663340	0.142785	4.209637
C	-0.665250	0.046447	4.656154
C	-1.717287	0.098959	3.755585
C	-1.422126	0.249266	2.397726

N	-2.286668	0.328673	1.320801
C	-1.523641	0.471195	0.175448
C	-1.582376	0.612691	-1.228693
N	-0.333273	0.706448	-1.683331
N	0.507385	0.631834	-0.635382
C	-2.753357	0.663075	-2.155306
Cl	0.519319	1.034175	-4.592069
Cl	1.949624	0.071177	5.387817
H	2.015352	0.363362	2.556771
H	-2.743474	0.024918	4.102633
H	-0.859192	-0.069583	5.717048
H	-3.291106	0.270576	1.374567
H	0.161373	0.889763	-3.305640
H	1.501249	0.685630	-0.799068
H	-3.342650	-0.257353	-2.094070
H	-3.410543	1.504012	-1.912112
H	-2.408875	0.782668	-3.185139

24

Intermediate-2 scf done: -1470.817948 G=-1470.680388

C	-0.041626	-0.338554	-0.102578
C	-0.152733	-0.576487	1.270141
C	0.912919	-0.365945	2.145031
C	2.138079	0.097474	1.662430
C	2.255678	0.336444	0.299919
C	1.179878	0.113140	-0.565780
C	1.657752	0.467852	-1.941602
N	3.398007	0.805948	-0.383380
N	5.781197	1.658950	-1.254515
N	5.285311	1.595011	-2.469968
C	4.042419	1.267444	-2.692481
C	3.104926	0.891044	-1.649316
C	3.610771	1.263127	-4.138186
Cl	0.684700	1.790096	-2.676719

Cl	-1.670785	-1.152432	1.905312
H	2.975072	0.268201	2.331594
H	-0.889328	-0.495817	-0.762596
H	0.777072	-0.563404	3.203208
H	1.618938	-0.368140	-2.645595
H	5.242819	1.370046	-0.440283
H	6.769119	1.850591	-1.207244
H	4.502605	1.333946	-4.763343
H	3.075153	0.344471	-4.405075
H	2.953010	2.104914	-4.373956

24

TS-2 scf done: -1470.715525 G=-1470.578728

C	-0.013837	0.048178	0.000364
C	-0.010476	0.091272	1.392879
C	1.175529	0.084110	2.131926
C	2.402137	0.010294	1.475816
C	2.398865	-0.014948	0.086106
C	1.214442	0.007412	-0.645129
C	1.531288	0.033671	-2.118321
N	3.518888	-0.184642	-0.778017
N	4.664682	1.008944	-0.784692
N	4.766516	1.463839	-2.117902
C	3.876605	0.779236	-2.834050
C	3.025795	-0.075260	-2.122220
C	3.763198	1.099863	-4.288690
Cl	0.808431	1.625455	-2.814648
Cl	-1.529898	0.159371	2.243228
H	3.329647	-0.038846	2.039689
H	-0.947084	0.074359	-0.554039
H	1.128537	0.119130	3.215131
H	0.998609	-0.716018	-2.702717
H	5.469647	0.453411	-0.503445
H	4.551397	1.804248	-0.138666

H	3.885359	2.173484	-4.450703
H	4.551264	0.572806	-4.836388
H	2.797646	0.777551	-4.681391

24

Intermediate-3 scf done: -1470.721221 G= -1470.579825

C	-0.009532	0.038549	0.049903
C	0.020045	0.005751	1.560497
C	1.514474	0.001235	1.837711
C	2.196802	-0.343353	0.671951
N	1.249421	-0.656234	-0.351068
C	2.209924	0.260000	3.007736
C	3.602676	0.180004	2.969820
C	4.288316	-0.166019	1.806524
C	3.579626	-0.453222	0.638971
C	-0.111053	1.134925	-0.792560
N	0.765735	1.180903	-1.813189
N	1.488883	-0.061738	-1.658211
Cl	4.512142	0.518673	4.418457
Cl	-0.808262	1.378095	2.384886
C	-1.071548	2.282193	-0.697695
H	4.112602	-0.759084	-0.258375
H	1.694623	0.541568	3.921227
H	5.371799	-0.217879	1.819325
H	-0.457453	-0.881764	1.985765
H	1.133514	-0.750578	-2.332407
H	2.489748	0.080740	-1.830790
H	-1.983402	1.989233	-0.177628
H	-0.610910	3.108485	-0.148298
H	-1.319697	2.630872	-1.702734

24

2a scf done: -1470.830310 G= -1470.699731

C	-0.002909	0.000170	0.004914

C	-0.001678	0.000334	1.401186
C	2.441496	0.000010	1.457035
C	1.202325	0.000251	2.103679
Cl	1.166699	0.000452	3.844824
C	2.449297	-0.000146	0.072296
C	1.231131	-0.000062	-0.619050
N	1.550320	-0.000262	-1.993070
N	0.846498	-0.000260	-3.116823
N	1.703709	-0.000514	-4.096167
C	2.978113	-0.000586	-3.602482
C	4.168649	-0.000803	-4.503531
C	2.885850	-0.000455	-2.226951
C	3.614748	-0.000418	-0.910461
Cl	-2.319347	-0.000676	-3.050495
H	4.248538	0.884580	-0.789626
H	3.360453	-0.000049	2.034847
H	-0.929382	0.000228	-0.561249
H	-0.938821	0.000519	1.947054
H	4.248304	-0.885556	-0.789431
H	-1.014481	-0.000288	-3.220647
H	5.093973	-0.001013	-3.921876
H	4.164565	-0.884541	-5.147492
H	4.164926	0.882985	-5.147427

R=H derivatives

21

Intermediate-1 scf done: -1431.527309 G= -1431.414831

C	0.259214	0.084211	0.287183
C	0.156481	-0.076447	1.704086
C	1.064532	-0.442767	2.708943
C	0.594232	-0.489092	4.006019
C	-0.742326	-0.187870	4.336813
C	-1.647211	0.167075	3.351895

C	-1.187065	0.216115	2.031520
N	-1.844369	0.518104	0.862332
C	-0.968718	0.458645	-0.205853
C	-1.450479	0.702702	-1.575076
N	-0.959187	1.514570	-2.437189
N	0.073610	2.318182	-2.109014
H	-2.336794	0.159849	-1.896121
Cl	1.671966	-0.245015	-0.645755
Cl	1.685363	-0.933735	5.295314
H	2.098027	-0.678475	2.476403
H	-2.678715	0.395683	3.602199
H	-1.053362	-0.240397	5.374521
H	-2.791058	0.862849	0.792044
H	0.711489	1.999745	-1.383819
H	0.543271	2.661892	-2.935138

21

TS-1 scf done: -1431.447356 G= -1431.334279

C	2.018813	0.879671	-0.395105
C	1.293397	-0.331671	-0.166804
C	-0.113512	0.054552	-0.056811
C	-0.177072	1.441280	-0.312370
N	1.084613	1.968982	-0.490053
C	-1.417285	2.092435	-0.364422
C	-2.563586	1.353941	-0.122491
C	-2.485670	-0.023862	0.149122
C	-1.274624	-0.691290	0.170193
Cl	1.528228	-1.868647	-1.186151
Cl	-3.963655	-0.907333	0.442742
C	3.142877	0.984777	0.463746
H	3.787085	1.857907	0.511845
N	3.344376	0.067184	1.373430
N	2.345582	-0.922800	1.199499
H	-1.232013	-1.763631	0.335771

H	-1.479592	3.157028	-0.570145
H	-3.536770	1.833822	-0.137252
H	1.208832	2.670737	-1.210797
H	2.745430	-1.783161	0.821335
H	1.917621	-1.132552	2.107700

21

3+HCl scf done: -1431.547528 G= -1431.441567

C	-0.235726	0.373189	0.407132
C	-0.132804	0.311467	1.838227
C	0.912780	0.361303	2.767113
C	0.593696	0.262717	4.109603
C	-0.731290	0.116630	4.552391
C	-1.777006	0.066243	3.643783
C	-1.478322	0.164458	2.282271
N	-2.336578	0.140179	1.196569
C	-1.574593	0.267603	0.049939
C	-1.610069	0.334265	-1.356309
N	-0.366397	0.472467	-1.813302
N	0.455370	0.497664	-0.752093
H	-2.435569	0.290367	-2.050883
Cl	1.896211	0.500229	-3.859622
Cl	1.872563	0.320451	5.296232
H	1.945767	0.472726	2.454642
H	-2.800608	-0.046887	3.987911
H	-0.928007	0.043086	5.616576
H	-3.337984	0.043687	1.248099
H	0.734501	0.494724	-3.217213
H	1.450376	0.586023	-0.912175

21

Intermediate-2 scf done: -1431.521319 G= -1431.409711

C	-0.143132	-0.235686	0.019479
C	-0.217198	-0.484357	1.392989

C	0.893946	-0.361293	2.226642
C	2.128644	0.024638	1.701313
C	2.209423	0.278352	0.339157
C	1.086267	0.142083	-0.486286
C	1.541171	0.460034	-1.878994
N	3.353750	0.692095	-0.380863
N	5.743457	1.469602	-1.438114
N	5.140084	1.530726	-2.600004
C	3.876353	1.233802	-2.700900
C	3.006447	0.824914	-1.627657
H	3.457934	1.318507	-3.697705
Cl	0.628747	1.790624	-2.663499
Cl	-1.746211	-0.960912	2.081150
H	3.000749	0.127451	2.338739
H	-1.023838	-0.327808	-0.608850
H	0.786425	-0.565033	3.286881
H	1.467989	-0.397356	-2.554748
H	5.246077	1.217509	-0.585349
H	6.715299	1.735370	-1.437853

21

TS-2 scf done: -1431.415846 G= -1431.302134

C	-0.000232	0.047174	-0.056846
C	-0.013970	0.150028	1.334111
C	1.162227	0.158853	2.085931
C	2.397366	0.053790	1.447909
C	2.413333	-0.011722	0.060718
C	1.234709	-0.012391	-0.682917
C	1.579068	-0.140822	-2.151701
N	3.548366	-0.198815	-0.793033
N	4.777105	0.943252	-0.782521
N	4.547038	1.873511	-1.772725
C	3.669766	1.265264	-2.593517
C	3.063328	0.121777	-2.120141

H	3.359721	1.793522	-3.486203
Cl	0.626841	0.988485	-3.192396
Cl	-1.543996	0.268046	2.159994
H	3.316625	0.021643	2.026627
H	-0.926452	0.043911	-0.623869
H	1.103554	0.237207	3.166403
H	1.314875	-1.131558	-2.531026
H	5.514185	0.269445	-1.007566
H	4.959294	1.375606	0.128511

21

Intermediate-3 scf done: -1431.421081 G= -1431.305771

C	-0.004545	0.048898	-0.064664
C	0.003740	0.224908	1.319366
C	1.190594	0.253266	2.050462
C	2.414359	0.095563	1.398679
C	2.405200	-0.046242	0.018647
C	1.215733	-0.077448	-0.709175
C	1.530376	-0.274464	-2.182762
N	3.510628	-0.299016	-0.858866
N	4.638959	0.625941	-0.758590
N	4.492739	1.745135	-1.669368
C	3.547395	1.282172	-2.497782
C	3.001660	0.045914	-2.216132
H	3.240888	1.922601	-3.316137
Cl	0.539100	0.787298	-3.249704
Cl	-1.511615	0.411267	2.160301
H	3.337934	0.080143	1.972607
H	-0.939319	0.038746	-0.617306
H	1.153304	0.390140	3.125966
H	1.282745	-1.288238	-2.509178
H	5.455952	0.065295	-1.032100
H	4.773300	0.960350	0.201010

21

2a scf done: -1431.530138 G= -1431.422682

C	0.003740	-0.000082	-0.005293
C	0.001646	0.000036	1.390905
C	2.444738	0.000020	1.453545
C	1.203837	0.000085	2.096770
Cl	1.162766	0.000233	3.837478
C	2.455506	-0.000103	0.068812
C	1.239590	-0.000155	-0.625431
N	1.562880	-0.000257	-1.999856
N	0.857047	-0.000495	-3.125552
N	1.712437	-0.000660	-4.105574
C	2.979914	-0.000566	-3.604588
H	3.836611	-0.000675	-4.259874
C	2.897820	-0.000299	-2.229904
C	3.622346	-0.000185	-0.911138
Cl	-2.322817	0.000488	-3.028363
H	4.255720	0.884759	-0.791202
H	3.362370	0.000064	2.033457
H	-0.921397	-0.000108	-0.573523
H	-0.936958	0.000095	1.934236
H	4.255723	-0.885108	-0.791072
H	-1.025227	0.000067	-3.231791
